# A conserved chaperone protein is required for the formation of a noncanonical type VI secretion system spike tip complex

**DOI:** 10.1016/j.jbc.2025.108242

**Published:** 2025-01-27

**Authors:** Kartik Sachar, Katarzyna Kanarek, Jake Colautti, Youngchang Kim, Eran Bosis, Gerd Prehna, Dor Salomon, John C. Whitney

**Affiliations:** 1 Department of Biochemistry and Biomedical Sciences, McMaster University, Hamilton, Ontario, Canada; 2 Department of Microbiology, University of Manitoba, Winnipeg, Manitoba, Canada; 3 Department of Clinical Microbiology and Immunology, Faculty of Medical and Health Sciences, Tel Aviv University, Tel Aviv, Israel; 4 Structural Biology Center, X-ray Science Division, Advanced Photon Source, Argonne National Laboratory, Lemont, Illinois, USA; 5 Department of Biotechnology Engineering, Braude College of Engineering, Karmiel, Israel; 6 Michael DeGroote Institute for Infectious Disease Research, McMaster University, Hamilton, Ontario, Canada

**Keywords:** type VI secretion system, bacterial competition, DUF2169, molecular chaperones, DUF4150, X-ray crystallography, *Pseudomonas aeruginosa*, *Vibrio parahaemolyticus*

## Abstract

Type VI secretion systems (T6SSs) are dynamic protein nanomachines found in Gram-negative bacteria that deliver toxic effector proteins into target cells in a contact-dependent manner. Prior to secretion, many T6SS effector proteins require chaperones and/or accessory proteins for proper loading onto the structural components of the T6SS apparatus. However, despite their established importance, the precise molecular function of several T6SS accessory protein families remains unclear. In this study, we set out to characterize the DUF2169 family of T6SS accessory proteins. Using gene co-occurrence analyses, we find that DUF2169-encoding genes strictly co-occur with genes encoding T6SS spike complexes formed by valine-glycine repeat protein G (VgrG) and DUF4150 domains. Although structurally similar to Pro-Ala-Ala-Arg (PAAR) domains, “PAAR-like” DUF4150 domains lack PAAR motifs and instead contain a conserved PIPY motif, leading us to designate them PIPY domains. Next, we present both genetic and biochemical evidence that PIPY domains require a cognate DUF2169 protein to form a functional T6SS spike complex with VgrG. This contrasts with canonical PAAR proteins, which bind VgrG on their own to form functional spike complexes. By solving the first crystal structure of a DUF2169 protein, we show that this T6SS accessory protein adopts a novel protein fold. Furthermore, biophysical and structural modeling data suggest that DUF2169 contains a dynamic loop that physically interacts with a hydrophobic patch on the surface of its cognate PIPY domain. Based on these findings, we propose a model whereby DUF2169 proteins function as molecular chaperones that maintain VgrG–PIPY spike complexes in a secretion-competent state prior to their export by the T6SS apparatus.

Many species of bacteria compete with other microbes to acquire resources from their environment (1, 2). One of the mechanisms used by Gram-negative bacteria to directly antagonize other Gram-negative bacteria is a cell envelope-embedded contractile nanomachine known as the type VI secretion system (T6SS) (3). The T6SS is a dynamic multiprotein complex that delivers toxic effector proteins to neighboring cells in a cell contact-dependent manner and thus confers a fitness advantage to strains that harbour a T6SS against susceptible competitors (4).

The T6SS is divided into three core subcomplexes: a membrane complex, a baseplate complex, and a bacteriophage tail-like complex (5, 6). The membrane complex anchors the T6SS in the diderm cell envelope of Gram-negative bacteria and forms a conduit through which effector proteins are exported from the cell (7). The baseplate complex exists in the cytoplasm and is thought to serve as a nucleation point for the polymerization of the phage tail-like component of the apparatus (8). Finally, the phage tail-like complex is structurally homologous to the tail component of contractile bacteriophages, but instead of interacting with a nucleic acid containing capsid, it serves as the effector delivery mechanism of the T6SS apparatus (9, 10, 11). The tail complex consists of several key proteins implicated in effector export: hemolysin-coregulated protein (Hcp), valine-glycine repeat protein G (VgrG), TssB, and TssC (5). Hcp forms hexameric rings that stack in a head-to-tail manner into a hollow tube (12). At one end of this tube exists a trimer of VgrG proteins that resemble a phage tail spike (13). TssB and TssC form a sheath that encloses the Hcp tube and current data suggests that the interaction of certain effector proteins with VgrG is a prerequisite for the contraction of TssB/TssC sheath (14, 15, 16). Upon sheath contraction, the Hcp-VgrG tube, along with any interacting effectors, is exported through the membrane complex and directly into a neighboring cell in a single firing event (17, 18).

Although a core set of 13 T6SS components were initially identified across many Gram-negative bacteria, the effector repertoire can differ significantly between different species or even strains of the same organism (3, 19, 20). These effectors are often encoded by gene clusters that are genomically distant from the core T6SS apparatus genes and often also encode an effector-specific VgrG and/or Hcp proteins, which allows multiple effector-containing tail–tube complexes to be exported by the same T6SS apparatus. The observation of this pattern of gene arrangement led to the identification of a 14th core T6SS component belonging to the Pro-Ala-Ala-Arg (PAAR) domain superfamily (14). Although sometimes found as individual proteins, PAAR domains frequently exist as N-terminal fusions to effector proteins, and as such, they facilitate the physical association of effectors with cognate VgrGs (21). Structurally, this is accomplished by the tight association of a single cone-shaped PAAR domain with the blunt tip of a VgrG trimer. When in complex with VgrGs, PAAR domains have been proposed to “sharpen” the T6SS spike so that it can penetrate the outer membrane of target cells (14). However, the distribution of PAAR proteins among T6SS-containing bacteria does not mirror that of VgrG proteins suggesting that in some cases, other protein families may exist that fulfill its role as an essential component of the T6SS spike. Indeed, a recent analysis by Zhang and colleagues identified the non-PAAR domain families DUF4280 and DUF4150 as proteins that share a similar predicted conical structure as PAAR but lack its namesake Pro-Ala-Ala-Arg motifs (22).

In addition to PAAR or PAAR-like domains, T6SS effector recruitment and loading onto the phage tail–tube complex often requires additional accessory proteins (23, 24, 25, 26). These accessory proteins belong to the DUF1795, DUF4123, and DUF2169 protein families, but our understanding of their precise molecular function varies. DUF1795 proteins function as chaperones that interact with the transmembrane domains (TMDs) of PAAR domain–containing membrane protein effectors, which allows them to stably exist in the cytoplasm while they are loaded onto their cognate VgrG (21, 27). DUF4123 proteins function as adaptor proteins that provide a direct physical connection between VgrG proteins and cognate effectors that lack PAAR or “PAAR-like” N-terminal domains (28, 29). By contrast, the molecular function of DUF2169 remains largely unexplored.

In this study, we employ a bioinformatics approach to identify a gene co-occurrence pattern between DUF2169 proteins and the DUF4150 “PAAR-like” protein family. We then demonstrate a genetic interaction between one such DUF2169-DUF4150 pair in *Vibrio parahaemolyticus*. To gain insight into DUF2169’s function, we solve the first X-ray crystal structure of this protein family, revealing a novel protein fold. Guided by this structure and predictive modeling of DUF4150, we then demonstrate that a direct physical interaction between DUF2169 and DUF4150 underlies their genetic interdependency. Overall, these findings significantly advance our understanding of the role played by an understudied accessory protein in T6SS-mediated effector delivery. Additionally, they reveal the existence of a widespread PAAR-like alternative spike tip structure.

## Results

### Genes encoding DUF2169, VgrG, and “PAAR-like” DUF4150 proteins are genetically linked

**Figure 1:**
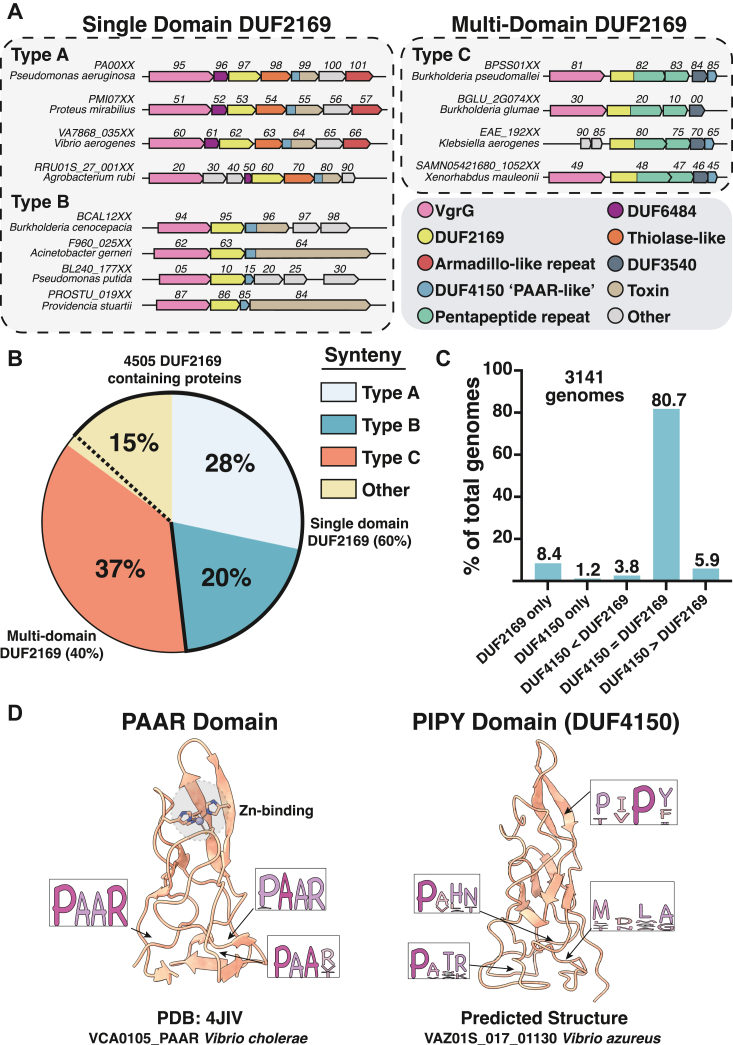
**DUF2169 co-occurs with DUF4150 “PAAR-like” proteins in three conserved gene syntenies**. *A*, genome neighborhood network of DUF2169-encoding genes, organized into three synteny categories. Synteny types A and B encode mostly single-domain DUF2169 proteins, whereas synteny type C encodes predominantly multidomain DUF2169 proteins with a C-terminal pentapeptide repeat protein (PRP) extension. *B*, distribution of DUF2169 synteny types across bacterial genomes. *Black outline* demarcates single domain from multidomain DUF2169 proteins. *C*, co-occurrence analysis of DUF2169 and DUF4150 proteins across 3141 genomes. *D*, structural model of the PAAR protein family, represented by *V*. *cholerae* VCA0105 (PDB: 4JIV), and the DUF4150 protein family, represented by the AF3-predicted PIPY domain of *V*. *azureus* VAZ01S_017_01130 (residues 1–137). Unlike PAAR domains, the PIPY domain lacks the PAAR motif and features a conserved P(I/V)P(Y/F) motif near the vertex of its conical structure. AF3, AlphaFold3.

Previous studies have anecdotally reported the co-occurrence of genes encoding DUF2169 proteins with other components of the T6SS including *vgrG*, effector, and so-called “PAAR-like” genes (23, 30, 31). Because gene co-occurrence in bacterial genomes often indicates a functional relationship between the encoded proteins, we set out to formally consolidate the totality of DUF2169-containing gene clusters using genome neighborhood analysis as a starting point for elucidating DUF2169 function. Our analysis revealed three predominant DUF2169 gene arrangements, referred to herein as synteny types A, B, and C (Fig. 1*A*. Each synteny type is characterized by the presence of distinct conserved domains in the genes located downstream of the DUF2169-containing gene: so-called “thiolase-like” in synteny type A, DUF4150 in synteny type B, and pentapeptide-repeat in synteny type C.

Additionally, our analysis led to the identification of two distinct types of DUF2169 gene products: single and multidomain proteins. We analyzed a total of 4505 genomes to determine the frequency of these gene syntenies and domain organization. Among all identified DUF2169 genes, approximately 60% are single domain, encoding only DUF2169. Of these single domains DUF2169, 28% of the total genes were classified within synteny type A gene clusters and 15% were within synteny type B gene clusters (Fig. 1*B*). The remaining 20% of single-domain DUF2169 genes were left uncategorized due to less defined gene synteny. Owing to the relatively simple arrangement of genes in synteny type B, we hypothesize that this cluster represents the minimal repertoire of T6SS components that function alongside DUF2169. Specifically, the only conserved co-occurring genes with DUF2169 in this syntenic arrangement are *vgrG* and genes encoding “PAAR-like” DUF4150 domains (InterPro accession: IPR025425).

Building on the minimal gene repertoire observed in synteny type B, synteny type A exhibits a more complex gene arrangement. In synteny type A, genes encoding DUF2169 proteins co-occur alongside three additional conserved genes of unknown function. Analysis of the protein products of these genes from *Proteus mirabilis* indicates that they belong to the DUF6484 (IPR045506), thiolase-like (IPR040771), and armadillo-like repeat (IPR006911) protein families. Although the precise roles of these proteins in T6SS are unknown, preliminary characterization of one synteny type A gene cluster in *P*. *mirabilis* using transposon mutagenesis showed that mutational inactivation of each gene within the cluster results in the loss of interbacterial killing of susceptible competitors by the DUF4150-containing PefE effector encoded within the cluster (32, 33). A second, partially characterized type A cluster exists in the model T6SS organism *Pseudomonas aeruginosa*, which expresses three differentially regulated and evolutionarily distinct T6SSs known as the H1-, H2-, and H3-T6SSs. In this bacterium, the genes encoding DUF2169 and its associated DUF4150-containing effector, Tse7, are genetically linked to the H1-T6SS (31). The H1-T6SS exports three VgrG spike proteins and characterization of Tse7-dependent interbacterial killing showed that it is entirely dependent on the synteny type A encoded VgrG1b protein (10, 11, 31, 34, 35). However, the potential involvement of DUF2169 or the additional three conserved synteny type A genes for Tse7 delivery has not been examined. Nonetheless, when taken together, these studies suggest that effectors encoded in synteny type A gene clusters likely require most if not all the conserved genes present in the cluster for their delivery into target cells.

In addition to the single-domain DUF2169 proteins encoded by synteny types A and B, approximately 37% of DUF2169-containing genes are found as multidomain proteins encoded within gene synteny type C (Fig. 1*B*). In these cases, the majority of DUF2169 proteins are fused to a predicted pentapeptide-repeat protein (PRP) domain at their C terminus (IPR001646). Like DUF2169 itself, the molecular function of the fused PRP domain is unknown. However, a recent report examining the rice plant-targeting T6SS of *Burkholderia glumae*, which contains a synteny type C DUF2169 gene, showed that both the N-terminal DUF2169 and the C-terminal PRP domains are essential for T6SS-dependent infection of rice plants (36). Therefore, it appears as though in the instances where this C-terminal PRP domain exists, it is equally important for T6SS function as its fused DUF2169 domain. As is the case with synteny types A and B, synteny type C also contains genes encoding VgrG and DUF4150 proteins. Finally, it uniquely contains a gene encoding a member of the uncharacterized DUF3540 (IPR021927) protein family, which has not been studied in any capacity. Overall, our informatics analyses of DUF2169 gene co-occurrence patterns indicate that although synteny type B likely represents the minimum functional unit required for the delivery of DUF2169-linked effectors into target cells, in instances where additional conserved genes are present, these too appear to be required for DUF2169-associated effector delivery.

Given the presence of genes encoding “PAAR-like” DUF4150 in all three synteny types, we next compared the relative abundance of these genes to that of DUF2169 genes within individual bacterial genomes. Similar to PAAR domains, DUF4150 domains occur as single domain proteins or as N-terminal domains of toxins as is the case with the aforementioned PefE and Tse7 effector proteins (Fig. S1*A*). In the vast majority of examined genomes, we found that genes harboring a DUF2169 domain are near or adjacent to genes containing a DUF4150 domain. By contrast, DUF2169 is rarely found near or adjacent to PAAR domain–containing genes (Fig. S1*B*). This analysis further revealed that in the majority of cases (>80%), DUF4150 and DUF2169 are present in a 1:1 ratio in bacterial genomes, suggesting that they may have a functional relationship with one another due to a physical interaction of the encoded proteins (Fig. 1*C*).

AlphaFold3 (AF3) confidently predicts that DUF4150 adopts the same overall conical shape of PAAR proteins despite lacking the namesake PAAR motifs and the conserved zinc-binding site found within the tip region of PAAR domains (Figs. 1*D* and S1*C*). In place of this internal zinc-binding site, DUF4150 possesses a hydrophobic core defined by a conserved “PIPY” motif. This hydrophobic core is conserved across all examined DUF4150 predicted structures and so to maintain consistency with PAAR domain nomenclature, we henceforth refer to DUF4150 domains as PIPY domains (Fig. S1*D*). The structural similarity between PIPY and PAAR domains strongly suggests that PIPY domains interact with VgrG in a manner that is analogous to the well-established spike–tip complexes formed by VgrGs and their cognate PAAR domains (14, 21, 24). However, in contrast to PIPY domains, there exists no DUF2169 equivalent subunit in PAAR–VgrG spike complexes that provides insight into DUF2169 function.

### DUF2169 is required for effector delivery in *P*. *aeruginosa* and *V*. *parahaemolyticus*

To directly assess the role of DUF2169 in effector delivery, we initiated bacterial competition experiments on the Tse7-linked DUF2169 protein encoded by the PA0097 locus of *P*. *aeruginosa*, which adopts a synteny type A gene arrangement. Using a previously described genetic background with constitutive H1-T6SS activity, we found that in contrast to the parent strain, which outcompetes Tse7-sensitive recipients by approximately 10-fold under our experimental conditions, a strain lacking DUF2169 exhibits no coculture fitness advantage and is indistinguishable from a control competition in which the donor strain lacks *tse7* (Fig. S2) (31). To ensure that this observed loss of competitive advantage is due to DUF2169 and not polar effects that result in inactivation of downstream genes, we next attempted to complement DUF2169 and restore Tse7-based killing in our DUF2169-deficient donor strain. However, despite using several orthogonal genetic strategies that reintroduced a WT copy of DUF2169 into *P*. *aeruginosa*, we were unable to restore the *tse7*-dependent fitness advantage exhibited by our parent strain. Thus, as is the case with prior transposon-based mutagenesis experiments in *P*. *mirabilis*, our results in *P*. *aeruginosa* are only suggestive of a requirement of DUF2169 for effector delivery.

**Figure 2:**
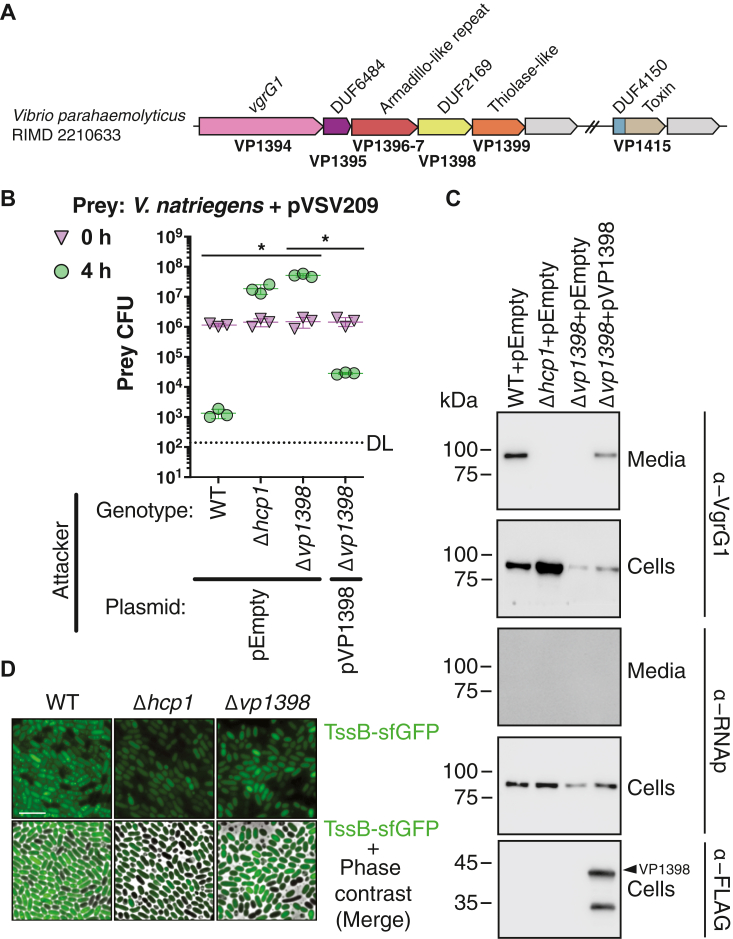
**A DUF2169 protein is required for T6SS1 activity in *V*. *parahaemolyticus*.***A*, genomic context of the *V*. *parahaemolyticus* T6SS1 DUF2169-encoding gene, *vp1398*. *B*, viability counts (colony forming units; CFUs) of *V*. *natriegens* prey strains containing an empty plasmid (pVSV209) for selection, before (0 h) and after (4 h) coincubation with the indicated *V*. *parahaemolyticus* attacker strain containing an empty plasmid (pEmpty) or a plasmid for the arabinose-inducible expression of VP1398 (pVP1398). The asterisk denotes statistical significance between samples at the 4 h time point calculated using an unpaired, two-tailed Student’s *t* test. Data are shown as the mean ± SD; n = 3 independent competition replicates. The data shown are a representative experiment out of three independent experiments. *C*, Expression (cells) and secretion (media) of VgrG1 from the indicated *V*. *parahaemolyticus* strains grown for 4 h at 30 °C in LB containing 3% [wt/vol] NaCl (MLB). RNA polymerase sigma 70 (RNAp) was used as a loading and lysis control. The expression of a C terminally FLAG-tagged VP1398 from a plasmid is shown in the *bottom panel*. Results from a representative experiment out of three independent experiments are shown. *D*, fluorescence microscopy of T6SS1 assembly in the indicated *V*. *parahaemolyticus* strains containing a plasmid for expressing a TssB1-sfGFP translational fusion. Representative images of TssB1-sfGFP signals are shown. The scale bar represents 5 μm. DL, the assay detection limit; T6SS, type VI secretion system.

Given our inconclusive results in *P*. *aeruginosa*, we next shifted our focus to *V*. *parahaemolyticus*, another DUF2169 encoding Gram-negative bacterium with a well-characterized T6SS. As is the case for *P*. *aeruginosa*, the genome of *V*. *parahaemolyticus* contains a single DUF2169 gene, *vp1398*, which is found in close genomic proximity to an experimentally validated effector gene encoding an N-terminal PIPY domain, *vp1415* (37, 38). Furthermore, like DUF2169 from *P*. *aeruginosa*, *V*. *parahaemolyticus* DUF2169 is encoded in a T6SS gene cluster (T6SS1) containing the conserved gene families that define the synteny type A arrangement (Fig. 2*A*). However, in contrast to the H1-T6SS of *P*. *aeruginosa*, which contains three semiredundant VgrG proteins that can each support overall T6SS apparatus function, the T6SS1 of *V*. *parahaemolyticus* only encodes a single VgrG spike protein, VgrG1 (11, 31, 38). Therefore, given the well-documented observation that T6SSs require at least one VgrG spike protein for function and our above informatics analyses that predict PIPY and DUF2169 proteins form a functional complex with a cognate VgrG spike, we hypothesized that loss of DUF2169 should mimic a T6SS1 deficient strain during bacterial competition. Indeed, in co-culture competition experiments against the T6SS1-susceptible bacterium, *Vibrio natriegens*, an attacker strain lacking DUF2169 exhibits a reduction in interbacterial killing that is comparable to that of a *V*. *parahaemolyticus* strain lacking the core T6SS1 subunit, Hcp1 (Fig. 2*B*). Importantly, this DUF2169-dependent loss of killing could be restored to near WT levels by plasmid-borne expression of DUF2169. Immunoblot analysis of these same strains revealed that the inability of the DUF2169 mutant to intoxicate *V. natriegens* is likely due to the DUF2169 protein being required for VgrG1 secretion (Fig. 2*C*). To support these results, TssB/C sheath assembly was used as an indicator of *V. parahaemolyticus* T6SS1 assembly (37, 39). In this experiment, TssB is translationally fused with sfGFP to observe sheath assembly. As shown in Fig. 2*D*, deletion of DUF2169 results in a complete loss of sheath assembly similar to the *hcp1* deletion control. Taken together with prior genetic analysis of the T6SS1 showing that deletion of the PIPY domain–containing VP1415 effector also abrogates T6SS1-dependent interbacterial killing (38), these data demonstrate a functional interaction between DUF2169, the PIPY-containing VP1415 effector, and *vgrG1*.

### The structure of a DUF2169 protein suggests a PIPY domain–binding site

To better understand why PIPY domains require DUF2169 proteins for function whereas structurally similar PAAR domains do not, we next undertook structural studies on VP1398. However, despite being able to express and purify milligram quantities of this protein, its crystallization proved refractory. To circumvent this obstacle, we next screened a panel of DUF2169 proteins encoded by other *Vibrio* spp. that are closely related to *V. parahaemolyticus* and found that the DUF2169 protein from *Vibrio xiamenensis*, which shares 64% pairwise sequence identity with DUF2169 from *V. parahaemolyticus*, readily crystallized and we were able to solve its X-ray crystal structure to a resolution of 1.85 Å (Table 1). The resulting electron density was of excellent quality, and we were able to model the majority of the 337 amino acid protein except for its three N-terminal and 12 C-terminal residues. The final model was refined to an *R*_work_ and *R*_free_ of 18.2% and 21.7%, respectively.

**Table 1:** X-ray data collection and structure refinement for *V. xiamenensis* DUF2169

Data collection	*V. xiamenensis* DUF2169
Wavelength (Å)	0.97918
Space group	P1
Cell dimensions	
*a*, *b*, *c* (Å)	53.35, 56.63, 69.51
*α*, *β*, *γ* (°)	83.27, 88.72, 66.72
Resolution (Å)	30.40–1.85 (1.90–1.85)
Unique reflections	53,211 (1298)
CC(1/2)	0.999 (0.629)
*R*_merge_ (%)	0.071 (1.325)
*I*/σ*I*	13.5 (1.3)
Completeness (%)	84.0 (40.9)
Redundancy	5.9 (5.6)
Refinement	
*R*_work_/*R*_free_ (%)	18.15/21.77
Average B-factors (Å^2^)	33.55
Ramachandran plot (%)	
Favored	97.19
Allowed	2.65
Outlier	0.16
PDB	8VTH

**Figure 3:**
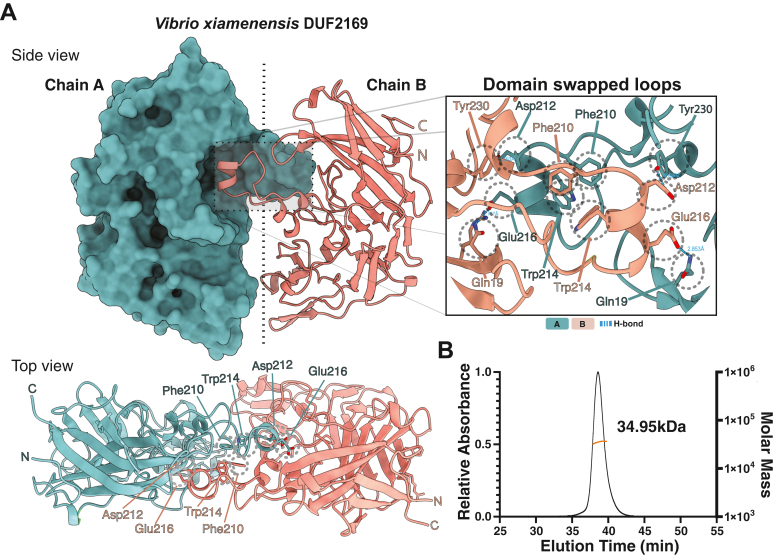
**X-ray crystal structure of DUF2169 from *Vibrio xiamenensis* reveals a domain-swapped homodimer.***A*, *V*. *xiamenensis* DUF2169 adopts a homodimeric arrangement mediated by a domain-swapped loop protruding from each protomer. Interacting residues of the domain-swapped loop are shown in *stick representation*, viewed from the side and top of the overall model. Close-up of the interacting residues is shown in the inset on the *right*. *B*, SEC-MALS chromatogram showing a single peak corresponding to calculated molecular weight (MW) of 34.95 kDa, indicating that *V*. *xiamenensis* DUF2169 is monomeric in solution (theoretical MW, 38.59 kDa). MALS, multiangle laser light scattering; SEC, size-exclusion chromatography.

The overall structure of *V*. *xiamenensis* DUF2169 reveals that its secondary structure is almost entirely comprised of β-strands arranged in three antiparallel β-sheets that form the core of the protein (Fig. 3*A*). These three β-sheets fold into two β-sandwiches that arise from the two shorter β-sheets stacking against the same face of the third, more elongated β-sheet. This arrangement results in the protein adopting a primarily flat, oblong structure with dimensions of approximately 77Å by ∼33Å by ∼31Å. The core of the protein is comprised of β-strands with the intervening loops forming the exterior of the protein. A search for structurally similar proteins in the Protein Data Bank using the DALI webserver indicates that the structure of DUF2169 is unique and therefore is considered a novel protein fold (40). Our structure also reveals that in its crystalline form, DUF2169 adopts a domain-swapped homodimeric arrangement with the swapped region (residues 195–232) consisting of a protruding loop that contains a short α-helix contributed by each protomer (Fig. 3*B*). The interface between the protomers is predominantly hydrophobic with several aromatic residues forming π-stacking interactions. In particular, Phe210 and Trp214 from one protomer π-stack with the equivalent residues from the other protomer. Additionally, the exterior of the domain-swapped α-helix positions Glu216 and Asp212 toward a shallow groove in the adjacent promoter where they form hydrogen bonds with Gln19 and Tyr230, respectively.

**Figure 4:**
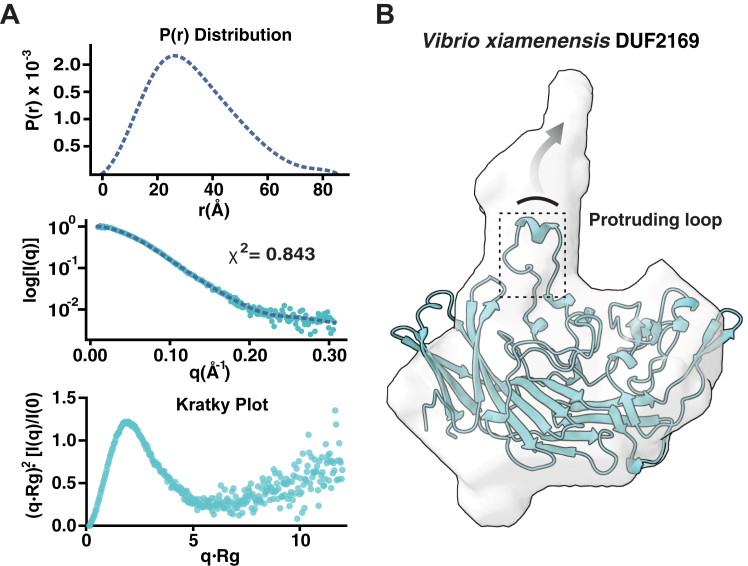
**Small-angle X-ray scattering analysis of *V*. *xiamenensis* DUF2169 indicates that it adopts a monomeric structure in solution.***A*, experimental dimensionless Kratky plot (*top*) demonstrates structural flexibility within DUF2169. P(r) distribution plot (*middle*) and data fit of P(r) distribution (*bottom*) indicating an extended protein, the corresponding scattering data was then used for the estimation of low-resolution electron density maps. *B*, SAXS-derived *ab initio* molecular envelope of *V*. *xiamenensis* DUF2169 fitted with a single protomer from the homodimeric X-ray crystal structure (chain A). SAXS, small-angle X-ray scattering.

To determine if the domain-swapped structure of DUF2169 observed in the crystal lattice reflects its oligomeric state in solution, we next performed size-exclusion chromatography coupled multiangle laser light scattering (SEC-MALS). Consistent with existing as a monomer in solution, SEC-MALS analysis revealed a single peak corresponding to a calculated molecular weight of 35.0 kDa, which closely aligns with the 38.6 kDa theoretical molecular weight of the *V*. *xiamenensis* DUF2169 polypeptide (Fig. 3*B*). To corroborate our SEC-MALS findings, we additionally examined both the molecular weight and overall shape of *V*. *xiamenensis* DUF2169 in solution using small-angle X-ray scattering (SAXS). Guinier analysis and subsequent real-space electron pair-wise distribution analysis (P(r)) of our SAXS data yielded radius of gyration (Rg) values of 25.9 Å and 25.4 Å, respectively (Fig. 4*A*, Table 2). These values are consistent with a molecular weight of ∼36 kDa, further bolstering our assertion that the physiological state of DUF2169 is monomeric. Perhaps most convincingly, SAXS analysis of VP1398 yielded near identical results indicating that both *V*. *xiamenensis* and *V*. *parahaemolyticus* DUF2169 proteins exist as monomers in solution (Fig. 3, Table 2).

**Table 2:** SAXS structural parameters from Guinier fits and P(r) functions using GNOM

Data collection	*V. xiamenensis* DUF2169 (5 mg ml^-1^)	*V. parahaemolyticus* DUF2169 (10 mg ml^-1^)
Guinier analysis		
*I*(0) (cm^-1^)	0.1 ± 1.18E-3	0.63 ± 0.01
R_g_ (Å)	25.87 ± 0.45	36.15 ± 0.73
*q*R_g_ max (Å^-1^)	1.279	1.32
*q*R_g_min (Å^-1^)	0.244	0.5
Coefficient of correlation, R^2^	0.975	0.91
P(r) analysis		
*I*(0) (cm^-1^)	0.102 ± 8.82E-03	0.53 ± 0.003
R_g_ (Å)	25.4 ± 0.22	29.12 ± 0.19
*d*_max_	85.0	91.0
*q* range (Å^-1^)	0.0094–0.3066	0.0137–0.366
*x*^2^ (total estimate from GNOM)	0.843 (0.884)	1.621 (0.84)
Corrected porod volume (V_p_) (Å^-3^) (ratio V_p_/calculated MW)	49,500 (1.2)	60,500 (1.2)
V_C_ MW (kDa) using Fischer method (ratio MW to expected)	36.5 (0.92)	41.0 (1.06)
MW using Bayesian (95% confidence interval)	36.9 (35.0–39.8)	44.7 (39.8–47.1)
DAMMIF/N		
*x*^2^	0.809	1.631
R_g_ (Å)	25.3	29.13
*d*_max_	106	96.3
Mean_NSD	0.964	2.02

SAXS, SAXS, small-angle X-ray scattering.

Our SAXS data also enabled the calculation of a low-resolution density map, which allowed us to assess the general shape of DUF2169 in solution (41, 42). In line with our molecular weight analyses, only one DUF2169 molecule fit into the calculated *ab initio* envelope and, interestingly, this high confidence fit resulted in a substantial region of the volume remaining unfilled near the protruding loop that participates in the domain-swapped crystallographic dimer (Fig. 4*B*). Due to its deviation from the ideal bell-shaped curve exhibited by well folded globular proteins, the dimensionless Kratky plot derived from our SAXS data indicates that a portion of DUF2169 is flexible and can adopt an unfolded state (Fig. 4*A*) (43). Based on its location near the unoccupied volume in our density map, our data support a model in which the protruding loop of DUF2169 is highly dynamic and, therefore, exists in multiple conformational states, some of which occupy the additional density observed in the SAXS molecular envelope. Collectively, these biophysical data support the conclusion that the domain-swapped dimer observed in our crystal structure is an artifact of crystal packing and that the protruding loop that facilitates this artifactual dimerization is highly dynamic and extends away from the core of the protein when it exists in its monomeric state in solution.

### The interaction between the PIPY domain of VP1415 and DUF2169 is required for T6SS1 function in *V*. *parahaemolyticus*

**Figure 5:**
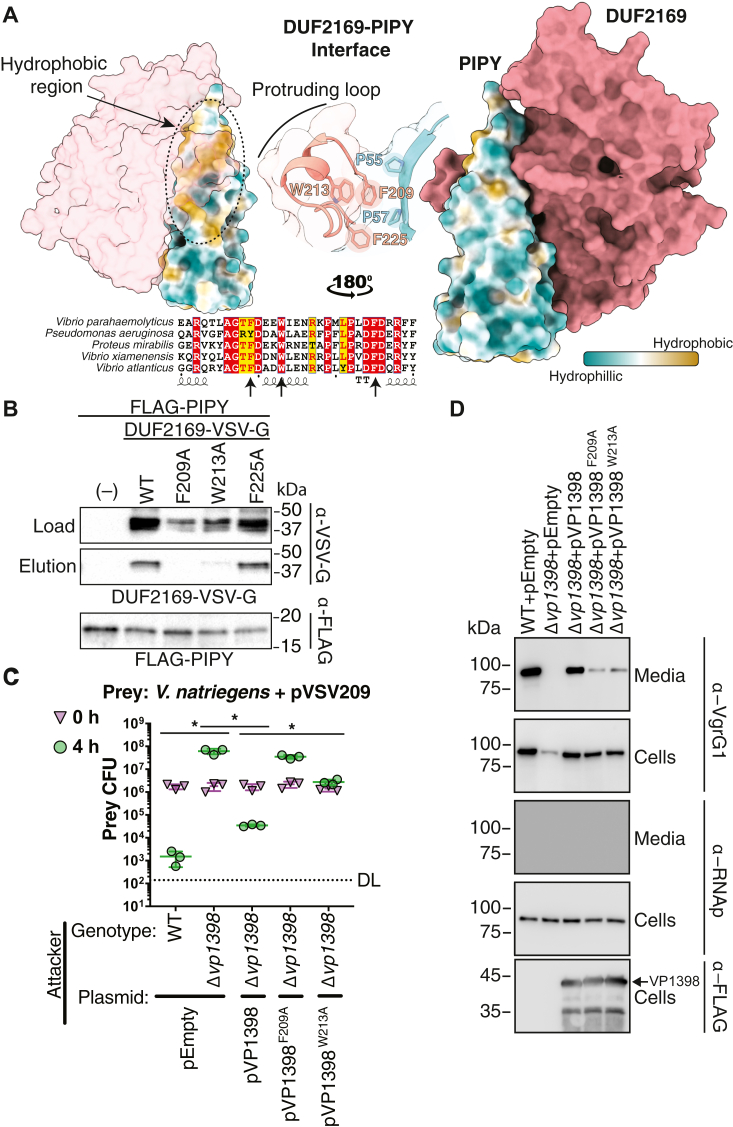
**DUF2169 interacts with the PIPY domain of its cognate effector in *V*. *parahaemolyticus* and this interaction is required for T6SS1-mediated interbacterial killing.***A*, a high-confidence complex prediction (mean pTM score > 90) of DUF2169 (VP1398) and PIPY (VP1415; residues 19–153) from *V*. *parahaemolyticus* was generated using AF3. The surface model of DUF2169 and hydrophobic surface of PIPY shows the P(I/V)P(Y/F) motif interacting with the protruding loop of DUF2169. Sequence alignment of the protruding loop of DUF2169 reveals that key residues are conserved across *V*. *parahaemolyticus* (Q87PV3), *P*. *aeruginosa* (Q9I735), *P*. *mirabilis* (B4EVA6), *V*. *xiamenensis* (A0A1G8DSF5), and *V*. *atlanticus* (B7VTT8), as indicated by their UniProt accessions. *B*, conserved *V*. *parahaemolyticus* DUF2169 (VP1398) protruding loop residues are required for binding its cognate PIPY domain (VP1415; residues 19–154). Western blot analysis of *E*. *coli* cells expressing VP1398 and the indicated site-specific mutants, each containing a C-terminal VSV-G tag, to assess their binding to a purified, N terminally FLAG-tagged VP1415 PIPY domain (residues 19–154). *C*, viability counts (colony-forming units; CFUs) of *V. natriegens* prey strains containing an empty plasmid (pVSV209) for selection, before (0 h) and after (4 h) coincubation with the indicated *V. parahaemolyticus* attacker strain containing an empty plasmid (pEmpty) or a plasmid for the arabinose-inducible expression of VP1398 (pVP1398) or its mutated forms. The statistical significance between samples at the 4 h time point was calculated using an unpaired, two-tailed Student’s *t* test; ns, no significant difference (*p* > 0.05). Data are shown as the mean ± SD; n = 3 independent competition replicates. The data shown are a representative experiment out of three independent experiments. *D*, expression (cells) and secretion (media) of VgrG1 from the indicated *V. parahaemolyticus* strains grown for 4 h at 30 °C in LB containing 3% [wt/vol] NaCl (MLB). RNA polymerase sigma 70 (RNAp) was used as a loading and lysis control. The expression of a C terminally FLAG-tagged VP1398 from a plasmid is shown in the *bottom panel*. Results from a representative experiment out of three independent experiments are shown. DL, the assay detection limit; LB, lysogeny broth; MLB, marine lysogeny broth; VgrG, valine-glycine repeat protein G.

We next used AF3 to assess the feasibility of a physical interaction occurring between DUF2169 and an AF3-predicted model of VP1415’s PIPY domain (44). Strikingly, the resulting complex is of very high confidence (ipTM of 0.89) and positions DUF2169 in such a way that it wraps around the PIPY domain in a manner that resembles fingers and a thumb grasping a cone (Fig. 5*A*). Similar complexes were also predicted when homologous proteins were used from closely related *V*. *xiamenensis* and more distantly related *P*. *mirabilis* (Fig. S4). Analysis of the predicted interface between PIPY and DUF2169 reveals that it is predominantly hydrophobic in nature and is formed by a prominent hydrophobic patch on the surface of the PIPY domain that interacts with a hydrophobic groove within DUF2169 (Fig. S4). Of particular interest given our biophysical data is the location of DUF2169’s dynamic protruding loop, which forms the thumb of the DUF2169–PIPY interaction and makes numerous contacts with residues found on the surface of PIPY. Based on this high confidence model, these findings suggest that DUF2169’s protruding loop participates in heterodimer formation in the presence of its physiological PIPY domain binding partner, whereas in its absence, nonphysiological homodimerization occurs at concentrations that likely exceed that achieved in the cell.

We next sought to validate our AlphaFold model using protein copurification analysis. Using differentially epitope-tagged proteins expressed in *Escherichia coli*, we found that the PIPY domain of VP1415 copurifies with its cognate DUF2169 protein (Fig. 5*B*). Given its dynamic properties in solution and its predicted role as the “thumb” region that wraps around the PIPY domain, we next examined the region of the interaction interface mediated by DUF2169’s protruding loop and identified Phe209, Trp213, and Phe225 as candidate residues mediating this interaction (Fig. 5*A*). Interestingly, these residues are predicted to interact with the two conserved proline residues found in the namesake PIPY motif that comprises the surface exposed hydrophobic patch that distinguishes PIPY domains from the more hydrophilic PAAR domains. Analysis of the distances between the centroid of each aromatic residue and the surface protruding C-H bonds of Pro55 and Pro57 from the PIPY motif suggest that Phe209 directly interacts with these residues *via* CH-π bonds, whereas the more distant Typ213 and Phe225 appear to be involved in maintaining the position of Phe209 rather than interacting with the abutting prolines directly (45). To test the importance of these DUF2169 residues for the interaction with the PIPY domain, we mutated each of them to alanine and examined the ability of the resulting site-specific variants to interact with VP1415’s PIPY domain *via* copurification. This analysis led to the identification of Phe209 and Trp213 as critical DUF2169 residues that are required for its interaction with PIPY (Fig. 5*B*). By contrast, the F225A variant of DUF2169 copurified with PIPY to the same levels as the wild-type protein.

**Figure 6:**
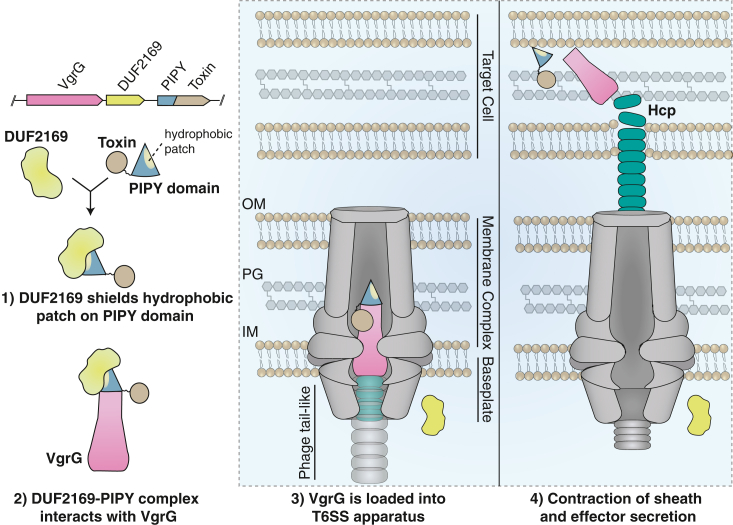
**Model of DUF2169-assisted loading of PIPY effectors into the T6SS.** Our data are suggestive of a model whereby the binding of DUF2169 to its cognate PIPY domain primes the effector for loading onto a VgrG spike protein. The DUF2169–PIPY effector complex is loaded onto VgrG prior to secretion, thus enabling the assembly of the T6SS tube, which subsequently leads to the killing of nonkin target cells. Schematic represents synteny type B; other syntenies are expected to function similarly except that synteny type A requires additional proteins, and synteny type C encodes the PIPY and effector domains as separate proteins. IM, inner membrane; OM, outer membrane; PG, peptidoglycan; T6SS, type VI secretion system; VgrG, valine-glycine repeat protein G.

Having identified variants of DUF2169 that no longer interact with PIPY, we next wanted to examine the consequences of disrupting this protein–protein interaction on interbacterial competition. To this end, we introduced our interaction-disrupting alleles of DUF2169 (VP1398) into *V*. *parahaemolyticus* Δ*vp1398* using our plasmid-based complementation system and competed the resulting strains against *V*. *natriegens*. In general agreement with our *in vitro* data, these experiments demonstrate that an attacking strain expressing VP1398^F209A^ exhibits a reduction in prey cell killing that is equivalent to that of an attacker lacking VP1398 altogether, whereas a strain expressing VP1398^W213A^ exhibits an intermediate killing phenotype, perhaps because Trp213 plays a more indirect role in the interaction interface compared to Phe209 (Fig. 5*C*). Finally, western blot analysis of T6SS1-dependent VgrG1 export indicates that the observed reduction in coculture fitness by *V*. *parahaemolyticus* strains expressing these DUF2169 variants is due to their substantially reduced secretion of VgrG1 (Fig. 5*D*). Taken together, our data indicate that DUF2169’s interaction with its cognate spike tip PIPY domain is required for the export of its genetically linked VgrG and the activity of *V*. *parahaemolyticus* T6SS1 (Fig. 6).

## Discussion

The complex network of protein–protein interactions required for T6SS function remains incompletely understood. In recent years, however, it has become apparent that the selective recruitment of effector proteins to the T6SS apparatus often requires the involvement of molecular chaperones that maintain effectors in a secretion competent state. Although T6SS proteins such as Hcp and Eag have been recognized as effector-stabilizing chaperones, the molecular role of the putative DUF2169 chaperone family has remained elusive (9, 23, 25, 27). Through the use of gene co-occurrence analysis, genetic and biochemical assays, and structural data, we show that DUF2169 is required for VgrG1 export and *V*. *parahaemolyticus* T6SS1 activity. Moreover, it physically interacts with VgrG1’s cognate PIPY domain and forms a tip complex that is distinct from characterized PAAR domains. Like PAAR domains, PIPY domains often harbor C-terminal toxin domains and therefore DUF2169 represents a critical link between T6SS toxins and VgrG spike proteins.

In contrast to their PAAR domain counterparts, PIPY domains feature a conserved hydrophobic surface containing their namesake PIPY motif in place of the canonical zinc-binding site (14). Interestingly, this hydrophobic surface comprises the protein–protein interaction interface between PIPY and the dynamic loop of DUF2169. This interaction is mediated through contacting proline-aromatic residues and we drew insights from the well-characterized Trp-cage miniproteins to explain this interaction. Similar to what we observe for our predicted DUF2169–PIPY complexes, aromatic–proline interactions in Trp-cage miniproteins are mediated through the negatively charged π face of the aromatic residue and the partially positively charged proline ring (46). CH–π bond formation requires a *cis-*proline, which, in the absence of an experimental structure of a DUF2169–PIPY complex, is difficult to predict. However, proline residues are likely to adopt a *cis-*conformation when encoded next to an aromatic residue (47). This offers a plausible explanation for the presence of a conserved Phe/Tyr adjacent to the interacting proline residues within the PIPY motif.

The exact function of the hydrophobic surface containing the PIPY motif remains unclear. However, its existence sets PIPY proteins apart from PAAR proteins, suggesting a unique role in the delivery of PIPY domain–containing effectors. In aqueous environments, such as the bacterial cytoplasm, surface-exposed hydrophobic residues are energetically unfavorable and can lead to protein aggregation. Therefore, we propose that the interaction of PIPY domains with their cognate DUF2169 protein prevents such aggregation during the trafficking of PIPY domain–containing toxins to the T6SS apparatus. A similar phenomenon has been observed for the small subset of PAAR domains that contain TMDs (24, 25). In this case, these proteins rely upon the Eag family of molecular chaperones to bind and stabilize these TMDs during the loading of the PAAR domain onto its cognate VgrG spike (21, 27, 48). Upon ejection of the PAAR–spike complex from the cell *via* the T6SS, Eag chaperones are thoughts to remain in the cytoplasm enabling the exposed TMDs to participate in membrane penetration of the target cell. Based on our findings herein, we propose a similar membrane-penetrating function for the hydrophobic surface of PIPY domains. In this case, DUF2169 proteins function to maintain PIPY domain–containing effectors in a secretion-competent state by shielding their hydrophobic surface prior to secretion. Upon export by the T6SS, the potential benefit of having such a hydrophobic spike protein could be the direct delivery of effectors into the target cell cytoplasm, a prediction that is supported by the association of both Eag chaperones and DUF2169 proteins with effectors possessing activities that function in the target cell cytoplasm (22). By contrast, effectors that act in the target cell periplasm may not require a spike tip protein with such hydrophobic properties.

The interaction of DUF2169 with the hydrophobic surface of PIPY domain–containing effectors may also prevent their erroneous insertion into the membranes of effector producing cells. If the role of the PIPY domain’s hydrophobic surface is inner membrane penetration of target cells, DUF2169 may play the role of a molecular “gun safety” that ensures that the T6SS-containing bacterium’s own membranes are not compromised from the cytoplasm. Although the mechanism for chaperone removal is not known for any T6SS chaperone family, having such a mechanism in place would enable rapid effector release upon target cell detection.

Collectively, our results provide evidence that DUF2169 proteins are required for the T6SS-dependent delivery of PIPY domain–containing effectors into target bacterial cells. Furthermore, our structural data suggests that DUF2169 functions as a chaperone for the hydrophobic region of PAAR-like domains. These newfound structural insights not only advance our understanding of T6SS structure and function but also provide new mechanistic details about the composition of T6SS warheads.

## Materials and methods

### Gene neighborhood analysis

Position-specific scoring matrices used in this study were downloaded from the NCBI Conserved Domain Database (49). A local database was constructed using all RefSeq genomes available from NCBI (last updated on August 21, 2023). RPS-BLAST was performed to identify DUF2169 homologs within this database, and results were filtered using an expect value threshold of 10 − 9. The analysis was restricted to complete genomes (NCBI assembly level: complete genome). The genomic neighborhoods of DUF2169-containing genes were analyzed as described previously (50, 51). Briefly, proteins encoded upstream and downstream of these genes were identified, and their sequences were examined using the Conserved Domain Database to detect conserved domains (49). Synteny types were defined by the presence of specific conserved domains in the gene located downstream of the DUF2169-containing gene. A comprehensive list of DUF2169-containing proteins and their adjacently encoded proteins is provided in Supplementary Data 1.

PAAR and PAAR-like proteins were identified following the same method used for DUF2169. This list was further refined to include only DUF4150 proteins. The corresponding list of DUF4150 proteins and adjacently encoded proteins is provided in Supplementary Data 2. Finally, a list of complete genomes containing either DUF2169 or DUF4150 was compiled and is available in Supplementary Data 3.

### Strains and media

*E*. *coli* strains was cultured in 2 × YT media (1.6% [w/v] tryptone, 1% [w/v] yeast extract, and 0.5% [w/v] NaCl) or on lysogeny broth (LB) agar plates (1.5% [w/v] agar, 1% [w/v] tryptone, 0.5% [w/v] yeast extract, and 0.5% [w/v] NaCl) at 37  °C. *P*. *aeruginosa* PAO1 cells were grown in LB media at 37 °C. *V*. *parahaemolyticus* and *V*. *natriegens* strains were grown in marine lysogeny broth (MLB; LB containing with 3% [w/v] NaCl) or on marine minimal medium (MMM) agar plates (1.5% [w/v] agar, 2% [w/v] NaCl, 0.4% [w/v] galactose, 5 mM MgSO_4_, 7 mM K_2_SO_4_, 77 mM K_2_HPO_4_, 35 mM KH_2_PO_4_, and 2 mM NH_4_Cl) at 30  °C. When required, media were supplemented with 10 μg/ml chloramphenicol and 50 or 250 μg/ml kanamycin (for *E*. *coli* and *Vibrio*, respectively) to maintain plasmids. Expression from the P*bad* promoters was induced by supplementing the media with 0.05% (w/v) L-arabinose. A list of all bacterial strains used in this study can be found in Table S1.

### Molecular cloning

Primers were synthesized and purified by Integrated DNA Technologies. A list of all primers used in this study can be found in Table S2. Phusion polymerase, restriction enzymes, and T4 DNA ligase were obtained from New England BioLabs. *E*. *coli* XL-1 Blue cells were used to transform and plasmid maintenance, SM10 for conjugal transfer of allelic exchange plasmids into *P*. *aeruginosa* PAO1, and BL21 Codon Plus for protein overexpression. Sanger sequencing was performed by Genewiz Incorporated. Primers for generating the pDM4 suicide vector and pBAD33.1-based vectors were synthesized by Sigma-Aldrich. DNA fragments were amplified *via* PCR from bacterial genomic DNA using ALLin Mega HiFi Mastermix Polymerase (highQu GmbH, HLM0201). The vectors and inserts were assembled using the Gibson Assembly Master Mix (NEB, E2611S). *E*. *coli* DH5α (λ-pir) cells were employed for transformation, plasmid maintenance, and tri-parental mating. A list of all plasmids used in this study can be found in Table S3.

### Construction of deletion strains

To generate an in-frame deletion of *vp1398* (VP_RS06790), 600 bp sequences flanking the upstream and downstream regions of the gene were subcloned into pDM4 plasmid (52). The resulting pDM4 construct was introduced into *V. parahaemolyticus* RIMD 2210633 *via* conjugation. Transconjugants were selected on MMM agar plates supplemented with chloramphenicol. To counter-select and ensure the loss of the SacB-containing pDM4 plasmid, transconjugants were subsequently grown on MMM agar plates containing 15% (w/v) sucrose. Deletions were confirmed by PCR.

Two-step allelic exchange was used to generate Δ*tse7-tsi7* and Δ*pa0097* gene deletion mutants as described by Schweizer (53). Briefly, 500 bp flanks downstream and upstream of the gene(s) being deleted were PCR amplified and then joined together using splicing by overlap extension PCR to create a ∼1 kb nucleotide product. This 1 kb product, which contains homologous regions surrounding the gene(s) being deleted, was ligated into the suicide vector pEXG2 (54). The resulting plasmid was then transformed into *E*. *coli* SM10 prior to being conjugated into *P*. *aeruginosa* PAO1 Δ*retS*, followed by selection of transconjugants using appropriate antibiotics. SacB based counter selection for mutants was achieved by growing cells on sucrose-containing solid media. Colonies harboring the desired deletion were identified by colony PCR.

### Protein overexpression and purification

DUF2169 from *V. parahaemolyticus* serotype O3:K6 (strain RIMD 2210633) and *V. xiamenensis* strain G21 were cloned into pET29b using standard restriction enzyme–based cloning. *E*. *coli* BL21 Codon Plus cells harboring these plasmids were grown to approximately an absorbance (λ = 600) of 0.6 in 1 L Fernbach flasks containing LB media (10 gL^−1^ NaCl, 10 gL^−1^ tryptone, and 5 gL^−1^ yeast extract) at 37 °C. They were then allowed to cool down to 18 °C before being induced with 1 mM IPTG. Cells were further grown overnight at 18 °C for approximately 16 h. Cells were centrifuged and resuspended in lysis buffer (50 mM Tris pH 8, 750 mM NaCl, 15 mM imidazole, and 5% glycerol). The resuspended cells were lysed using a sonicator at 30Amp for six cycles of 30 s. Lysed cells were centrifuged, and the supernatant was passed over expanded beads nickel-column for Ni-affinity chromatography. The beads were washed with 25 column volume of lysis buffer before being eluted using elution buffer (50 mM Tris–HCl pH 8.0, 750 mM NaCl, 400 mM imidazole, 1 mM 2-mercaptoethanol, 5% glycerol, and 400 mM imidazole). Protein samples were further purified by SEC using a HiLoad 16/600 Superdex200 or HiLoad 16/600 Superdex75 preparatory grade column. The final SEC buffer was 50 mM Tris–HCl pH 8.0, 750 mM NaCl, 5% glycerol, and 1 mM DTT.

### Protein crystallization

*V*. *xiamenensis* DUF2169 (SAMN04488136_12145) was concentrated to 15 mg ml^−1^ for initial screening using commercially available screens (MCSG1-4 suites, Anatrace) by sitting-drop vapour diffusion using a Crystal Gryphon robot. The initial crystallization conditions were 15 mg mL^−1^ protein concentration with a 1:1 mixture of 0.2 M calcium acetate and 25% (v/v) PEG 3350 at 4 °C. The microcrystals were extracted and crushed using the Seed Beads kit from Hampton Research. These crystal seeds (diluted 1:100) were added to varying concentrations of calcium acetate *versus* PEG 3350 in a hanging-drop vapour diffusion screen. Crystals obtained from 0.75 mM calcium acetate and 22% (v/v) PEG 3350 underwent a second round of seeding using the Seed Beads. Protein samples (15 mg ml^−1^) containing crystal seeds (diluted 1:100) were added to 48-well hanging-drop vapor diffusion screens. Commercially available Additive Screen (Hampton Research) and 0.75 mM calcium acetate and 22% (v/v) PEG 3350 were tested for improved crystal morphology and size. Improved crystals obtained from 0.75 mM calcium acetate, 22% (v/v) PEG 3350, and 2% benzamidine HCl were further optimized using macro-seeding *ex situ.* Intact crystals were extracted from the previous hanging drop and placed into a fresh hanging drop containing crystallization condition 0.75 mM calcium acetate, 22% (v/v) PEG 3350, and 2% benzamidine HCl and 15 mg ml^−1^ protein in a 1:1 mixture. This process was repeated until large enough crystals were obtained for diffraction.

Diffraction data was collected at 100 K on the Pilatus3 X 6M detector (dectris) at 19-ID beamline of the Structural Biology Center at the Advanced Photon Source. The data were processed using xia2 (55). The structure was solved using MOLREP implemented in HKL3000 with the AlphaFold2 model of DUF2169 (56, 57, 58). The structure was initially refined using refmac5.5 and manually adjusted using Coot (59, 60). The iterative refinement for the structure was followed using Phenix.refine with translation/libration/screw (TLS) parameterization for B factors and Coot until the structure converged to *R*_work_/*R*_free_ values of 0.18/0.22 with reasonable stereochemistry validated by Molprobity, PROCHECK, and PDB validation (61, 62, 63, 64). Throughout the refinement the same selected 5% data were removed and only used for *R*_free_ calculations which were used for monitoring the progress of the refinement. The final structure was deposited with the PDBID of 8VTH. See Table S1 for details. Figures were generated using ChimeraX (65).

### SEC with multiangle light scattering

SEC-MALS experiments were performed using buffer conditions similar to those used for standard SEC purification (50 mM Tris–HCl, pH 8.0, 750 mM NaCl, and 1 mM DTT). SEC was conducted using a Superdex 200 Increase 10/300 column (GE Healthcare), and MALS was carried out using a MiniDAWN and Optilab system (Wyatt Technologies). Data analysis was performed using the Astra software package (https://www.wyatt.com/products/software/astra.html).

### Small-angle X-ray scattering

SAXS was performed on the DUF2169 from *V*. *parahaemolyticus* (VP1398) and DUF2169 from *V*. *xiamenensis* (SAMN04488136_12145). Protein was purified using the procedure as described above and concentrated to 10 mg ml^-1^ protein in SEC buffer (50 mM Tris pH 8, 500 mM NaCl, 5% glycerol, and 1 mM DTT). SAXS diffractions were collected with a BioSAXS-1000 Rigaku detector. Software SAXSLab 3.1.0 was used for data collection. BioXTas Raw (https://github.com/jbhopkins/bioxtasraw) software was used for Guinier analysis and pair-distance distribution data processing (66). *Ab initio* model was built using DAMMIF *via* RAW with these parameters: 20 number of reconstructions, slow mode, P1 symmetry, unknown anisometry, and refinement with DAMMIN. The resulting PDB file was converted into a volume map using ChimeraX molmap function with a resolution of 5Å (65). The AF3 model of VP1398 was fit into the map using ChimeraX fit to map function with auto rotate and shift model into the map.

### Secretion assays

To monitor VgrG1 secretion, the indicated *Vibrio* strains were grown overnight in MLB and then normalized to an OD_600_ of 0.18 in 5 ml of fresh MLB supplemented with chloramphenicol, L-arabinose, and 20 μM phenamil (67). Cultures were incubated at 30 °C with constant shaking (220 rpm) for 3.5 h. For expression fraction analysis (cells), samples equivalent to 0.5 OD_600_ units were collected, and cell pellets were resuspended in 50 μl of 2x Tris-glycine SDS sample buffer (Novex, Life Sciences, LC2676). For secretion fraction analysis (media), supernatant volumes equivalent to 10 OD_600_ units were filtered through 0.22 μm filters, and proteins were precipitated using the deoxycholate and trichloroacetic acid method (68). Precipitated proteins were washed twice with cold acetone, air-dried, and resuspended in 20 μl of 100 mM Tris-Cl (pH 8.0) and 20 μl of 2x protein sample buffer containing 5% (v/v) β-mercaptoethanol.

### Western blotting

Western blot analyses of heterologous expressed proteins in *E*. *coli* were performed using standard SDS-PAGE gel and buffer system. After SDS-PAGE separation, proteins were wet-transferred to 0.45 μm nitrocellulose membranes (100 V for 30 min, 4 °C). The nitrocellulose membrane was incubated with 5% (w/v) blotting grade blocker (Bio-Rad) for either 2 h at 37 °C or overnight at 4 °C. The membrane was washed 3x with Tris-buffered saline + 5% Tween. The membrane was then incubated with tag-specific rabbit primary antibodies either ⍺-His, ⍺-FLAG, or ⍺-VSVG (1:5000, 1 h). The membrane was washed again 3x with Tris-buffered saline + 5% Tween and then incubated with secondary antibodies either ⍺-mouse (1:5000; primary ⍺-His) or ⍺-rabbit (1:5000; primary ⍺-FLAG/⍺-VSVG) for 30 min. Western blots were developed using a chemiluminescent substrate (Bio-Rad) and imaged using Chemi-Doc Imaging System (Bio-Rad).

For protein samples obtained from *Vibrio* secretion assays, samples were incubated at 95 °C for 10 min and resolved on TGX Stain-Free gels (Bio-Rad). Proteins were transferred onto 0.2 μm nitrocellulose membranes using the Trans-Blot Turbo Transfer System (Bio-Rad) according to the manufacturer’s protocol. Membranes were immunoblotted with α-FLAG (Sigma-Aldrich, F1804), Direct-Blot horseradish peroxidase anti-*E*. *coli* RNA polymerase sigma 70 (BioLegend, mouse mAb #663205, referred to as α-RNAp), or custom-made α-VgrG1 antibodies (69), used at 1:1000 dilution. Protein signals were visualized using the Fusion FX6 imaging system (Vilber Lourmat) and ECL reagents.

### Bacterial competition assays

For *Vibrio* competition assays, the indicated attacker and prey strains were grown overnight in MLB and normalized to an OD_600_ of 0.5. The strains were then mixed in triplicate at a 4:1 (attacker:prey) ratio. The mixtures were spotted (25 μl) onto MLB agar competition plates supplemented with L-arabinose. The plates were incubated at 30 °C for 4 h. To determine the initial colony-forming units (CFUs) of the prey strains at t = 0 h, 10-fold serial dilutions of the mixtures were plated on prey selective media. After 4 h of coincubation on the competition plates, the bacteria in each spot were harvested into 1 ml LB, and the CFUs of the surviving prey strains were quantified by plating 10-fold serial dilutions on prey selective media. Data visualization was performed using GraphPad Prism v9 (https://www.graphpad.com).

A tetracycline-resistant, *lacZ*-expression cassette was inserted into a neutral phage attachment site (*attB*) of recipient *P*. *aeruginosa* PAO1 for blue-white cell screening. Recipient and donor cells were grown overnight night in LB media at 37 °C. The next day, the two cultures were OD-matched and mixed at a 1:1 (v/v) ratio. The initial count of donors:recipients were determined by plating the mixture at the correct dilution on a solid 1.5% LB agar plate supplemented with 40 μg ml^−1^ X-gal. The competition mixture was spot plated on 0.45 μm nitrocellulose membrane overlaid on a 3% LB agar plate and incubated face up at 37 °C for 20 to 24 h. Competitions were then harvested by scraping cells off the agar plate and resuspending in LB. As before the final ratio of donors:recipients were determined by plating on LB agar containing 40 μg ml^−1^ X-gal. The final ratio of donors:recipients CFUs were normalized to the initial ratios of donor and recipient strains. Complementation was performed identically with the exception that all media were supplemented with 0.5 mM IPTG.

### Microscopy imaging

*V*. *parahaemolyticus* RIMD 2210633 WT, Δ*hcp1*, and Δ*vp1398* strains harboring the plasmid pTssB1-sfGFP, which enables arabinose-inducible expression of the T6SS1 sheath protein TssB1 (VP_RS06810) fused to sfGFP, were grown overnight in MLB supplemented with kanamycin to maintain the plasmids. Bacterial cultures were diluted 1:9 into fresh MLB-containing kanamycin, L-arabinose, and 20 μM phenamil, and then incubated for 2 h at 30 °C. Following incubation, 2 μl of the bacterial suspensions were spotted onto MLB-agarose pads (1.5% w/v) supplemented with L-arabinose and kanamycin. The pads were air-dried for 5 min and placed face-down in 35 mm glass-bottom Cellview cell culture dishes. Microscopy was performed using a Nikon Eclipse Ti2-E inverted motorized microscope equipped with a CFI Plan Apochromat DM 100 × oil lambda PH-3 (NA 1.45) objective lens, a Lumencor SOLA SE II 395 light source, and ET-EGFP (#49002) filter sets for sfGFP visualization. Images were captured with a DS-QI2 Mono cooled digital microscope camera (16 MP) and processed using Fiji (https://imagej.net/software/fiji/).

### Protein alignment, structure prediction, and visualization

All sequence alignments were performed using Omega Clustal (70) and visualized using ESPript 3.0 (71). All structural predictions were made using AF3 (44). All structural alignment and visualization were made using ChimeraX (65).

## Data availability

The X-ray structure of *V*. *xiamenensis* DUF2169 has been deposited in the Protein Data Bank under accession code 8VTH.

## Supporting information

This article contains supporting information (9, 39, 52, 72, 73, 74, 75, 76).

## Conflict of interest

The authors declare that they have no conflicts of interest with the contents of this article.

## References

[1] Hibbing M.E., Fuqua C., Parsek M.R., Peterson S.B. (2010). Bacterial competition: surviving and thriving in the microbial jungle. Nat. Rev. Microbiol..

[2] Peterson S.B., Bertolli S.K., Mougous J.D. (2020). The central role of interbacterial antagonism in bacterial Life. Curr. Biol. : CB.

[3] Coulthurst S. (2019). The Type VI secretion system: a versatile bacterial weapon. Microbiology (Reading).

[4] Russell A.B., Peterson S.B., Mougous J.D. (2014). Type VI secretion system effectors: poisons with a purpose. Nat. Rev. Microbiol..

[5] Silverman J.M., Brunet Y.R., Cascales E., Mougous J.D. (2012). Structure and regulation of the type VI secretion system. Annu. Rev. Microbiol..

[6] Cherrak Y., Flaugnatti N., Durand E., Journet L., Cascales E. (2019). Structure and activity of the type VI secretion system. Microbiol. Spectr..

[7] Durand E., Nguyen V.S., Zoued A., Logger L., Pehau-Arnaudet G., Aschtgen M.S. (2015). Biogenesis and structure of a type VI secretion membrane core complex. Nature.

[8] Cherrak Y., Rapisarda C., Pellarin R., Bouvier G., Bardiaux B., Allain F. (2018). Biogenesis and structure of a type VI secretion baseplate. Nat. Microbiol..

[9] Silverman J.M., Agnello D.M., Zheng H., Andrews B.T., Li M., Catalano C.E. (2013). Haemolysin coregulated protein is an exported receptor and chaperone of type VI secretion substrates. Mol. Cel..

[10] Hachani A., Allsopp L.P., Oduko Y., Filloux A. (2014). The VgrG proteins are "a la carte" delivery systems for bacterial type VI effectors. J. Biol. Chem..

[11] Whitney J.C., Beck C.M., Goo Y.A., Russell A.B., Harding B.N., De Leon J.A. (2014). Genetically distinct pathways guide effector export through the type VI secretion system. Mol. Microbiol..

[12] Ballister E.R., Lai A.H., Zuckermann R.N., Cheng Y., Mougous J.D. (2008). In vitro self-assembly of tailorable nanotubes from a simple protein building block. Proc. Natl. Acad. Sci. USA.

[13] Douzi B., Spinelli S., Blangy S., Roussel A., Durand E., Brunet Y.R. (2014). Crystal structure and self-interaction of the type VI secretion tail-tube protein from enteroaggregative Escherichia coli. PloS one.

[14] Shneider M.M., Buth S.A., Ho B.T., Basler M., Mekalanos J.J., Leiman P.G. (2013). PAAR-repeat proteins sharpen and diversify the type VI secretion system spike. Nature.

[15] Renault M.G., Zamarreno Beas J., Douzi B., Chabalier M., Zoued A., Brunet Y.R. (2018). The gp27-like hub of VgrG serves as adaptor to promote hcp tube assembly. J. Mol. Biol..

[16] Cianfanelli F.R., Alcoforado Diniz J., Guo M., De Cesare V., Trost M., Coulthurst S.J. (2016). VgrG and PAAR proteins define distinct versions of a functional type VI secretion system. PLoS Pathog..

[17] Russell A.B., Hood R.D., Bui N.K., LeRoux M., Vollmer W., Mougous J.D. (2011). Type VI secretion delivers bacteriolytic effectors to target cells. Nature.

[18] Basler M., Pilhofer M., Henderson G.P., Jensen G.J., Mekalanos J.J. (2012). Type VI secretion requires a dynamic contractile phage tail-like structure. Nature.

[19] Barret M., Egan F., Fargier E., Morrissey J.P., O'Gara F. (2011). Genomic analysis of the type VI secretion systems in Pseudomonas spp.: novel clusters and putative effectors uncovered. Microbiology (Reading).

[20] Geller A.M., Shalom M., Zlotkin D., Blum N., Levy A. (2024). Identification of type VI secretion system effector-immunity pairs using structural bioinformatics. Mol. Syst. Biol..

[21] Quentin D., Ahmad S., Shanthamoorthy P., Mougous J.D., Whitney J.C., Raunser S. (2018). Mechanism of loading and translocation of type VI secretion system effector Tse6. Nat. Microbiol..

[22] Zhang Z., Liu Y., Zhang P., Wang J., Li D., Li Y.Z. (2021). PAAR proteins are versatile clips that enrich the antimicrobial weapon arsenals of prokaryotes. mSystems.

[23] Bondage D.D., Lin J.S., Ma L.S., Kuo C.H., Lai E.M. (2016). VgrG C terminus confers the type VI effector transport specificity and is required for binding with PAAR and adaptor-effector complex. Proc. Natl. Acad. Sci. USA.

[24] Whitney J.C., Quentin D., Sawai S., LeRoux M., Harding B.N., Ledvina H.E. (2015). An interbacterial NAD(P)(+) glycohydrolase toxin requires elongation factor Tu for delivery to target cells. Cell.

[25] Alcoforado Diniz J., Coulthurst S.J. (2015). Intraspecies competition in serratia marcescens is mediated by type VI-Secreted Rhs effectors and a conserved effector-associated accessory protein. J Bacteriol..

[26] Liang X., Moore R., Wilton M., Wong M.J., Lam L., Dong T.G. (2015). Identification of divergent type VI secretion effectors using a conserved chaperone domain. Proc. Natl. Acad. Sci. USA.

[27] Ahmad S., Tsang K.K., Sachar K., Quentin D., Tashin T.M., Bullen N.P. (2020). Structural basis for effector transmembrane domain recognition by type VI secretion system chaperones. eLife.

[28] Unterweger D., Kostiuk B., Otjengerdes R., Wilton A., Diaz-Satizabal L., Pukatzki S. (2015). Chimeric adaptor proteins translocate diverse type VI secretion system effectors in Vibrio cholerae. EMBO J..

[29] Colautti J., Tan H., Bullen N.P., Thang S.S., Hackenberger D., Doxey A.C. (2024). A widespread accessory protein family diversifies the effector repertoire of the type VI secretion system spike. Nat. Commun..

[30] Bayer-Santos E., Ceseti L.M., Farah C.S., Alvarez-Martinez C.E. (2019). Distribution, function and regulation of type 6 secretion systems of Xanthomonadales. Front. Microbiol..

[31] Pissaridou P., Allsopp L.P., Wettstadt S., Howard S.A., Mavridou D.A.I., Filloux A. (2018). The Pseudomonas aeruginosa T6SS-VgrG1b spike is topped by a PAAR protein eliciting DNA damage to bacterial competitors. Proc. Natl. Acad. Sci. USA.

[32] Alteri C.J., Himpsl S.D., Pickens S.R., Lindner J.R., Zora J.S., Miller J.E. (2013). Multicellular bacteria deploy the type VI secretion system to preemptively strike neighboring cells. PLoS Pathog..

[33] Alteri C.J., Himpsl S.D., Zhu K., Hershey H.L., Musili N., Miller J.E. (2017). Subtle variation within conserved effector operon gene products contributes to T6SS-mediated killing and immunity. PLoS Pathog..

[34] Hachani A., Lossi N.S., Hamilton A., Jones C., Bleves S., Albesa-Jove D. (2011). Type VI secretion system in Pseudomonas aeruginosa: secretion and multimerization of VgrG proteins. J. Biol. Chem..

[35] Hood R.D., Singh P., Hsu F., Guvener T., Carl M.A., Trinidad R.R. (2010). A type VI secretion system of Pseudomonas aeruginosa targets a toxin to bacteria. Cell host & microbe.

[36] Kim N., Han G., Jung H., Lee H.H., Park J., Seo Y.S. (2021). T6SS accessory proteins, including DUF2169 domain-containing protein and pentapeptide repeats protein, Contribute to bacterial virulence in T6SS Group_5 of Burkholderia glumae BGR1. Plants (Basel).

[37] Ben-Yaakov R., Salomon D. (2019). The regulatory network of Vibrio parahaemolyticus type VI secretion system 1. Environ. Microbiol..

[38] Salomon D., Kinch L.N., Trudgian D.C., Guo X., Klimko J.A., Grishin N.V. (2014). Marker for type VI secretion system effectors. Proc. Natl. Acad. Sci. USA.

[39] Jana B., Keppel K., Salomon D. (2021). Engineering a customizable antibacterial T6SS-based platform in Vibrio natriegens. EMBO Rep..

[40] Holm L. (2020). Using Dali for protein structure Comparison. Methods Mol. Biol..

[41] Franke D., Svergun D.I. (2009). DAMMIF, a program for rapid ab-initio shape determination in small-angle scattering. J. Appl. Crystallogr..

[42] Svergun D.I. (1999). Restoring low resolution structure of biological macromolecules from solution scattering using simulated annealing. Biophysical J..

[43] Kikhney A.G., Svergun D.I. (2015). A practical guide to small angle X-ray scattering (SAXS) of flexible and intrinsically disordered proteins. FEBS Lett..

[44] Abramson J., Adler J., Dunger J., Evans R., Green T., Pritzel A. (2024). Accurate structure prediction of biomolecular interactions with AlphaFold 3. Nature.

[45] Xiao Y., Woods R.J. (2023). Protein-ligand CH-pi interactions: structural informatics, Energy function Development, and Docking Implementation. J. Chem. Theor. Comput.

[46] Neidigh J.W., Fesinmeyer R.M., Andersen N.H. (2002). Designing a 20-residue protein. Nat. Struct. Biol..

[47] Zondlo N.J. (2013). Aromatic-proline interactions: electronically tunable CH/pi interactions. Acc. Chem. Res..

[48] Jurenas D., Rosa L.T., Rey M., Chamot-Rooke J., Fronzes R., Cascales E. (2021). Mounting, structure and autocleavage of a type VI secretion-associated Rhs polymorphic toxin. Nat. Commun..

[49] Marchler-Bauer A., Bo Y., Han L., He J., Lanczycki C.J., Lu S. (2017). CDD/SPARCLE: functional classification of proteins via subfamily domain architectures. Nucleic Acids Res..

[50] Dar Y., Salomon D., Bosis E. (2018). The antibacterial and anti-Eukaryotic type VI secretion system MIX-effector repertoire in vibrionaceae. Mar. Drugs.

[51] Jana B., Salomon D., Bosis E. (2020). A novel class of polymorphic toxins in Bacteroidetes. Life Sci. Alliance.

[52] O'Toole R., Milton D.L., Wolf-Watz H. (1996). Chemotactic motility is required for invasion of the host by the fish pathogen Vibrio anguillarum. Mol. Microbiol..

[53] Schweizer H.P. (1992). Allelic exchange in Pseudomonas aeruginosa using novel ColE1-type vectors and a family of cassettes containing a portable oriT and the counter-selectable Bacillus subtilis sacB marker. Mol. Microbiol..

[54] Rietsch A., Vallet-Gely I., Dove S.L., Mekalanos J.J. (2005). ExsE, a secreted regulator of type III secretion genes in Pseudomonas aeruginosa. Proc. Natl. Acad. Sci. USA.

[55] Winter G., Lobley C.M., Prince S.M. (2013). Decision making in xia2. Acta Crystallogr. Section D, Biol. Crystallogr..

[56] Vagin A., Teplyakov A. (2010). Molecular replacement with MOLREP. Acta Crystallogr. Section D, Biol. Crystallogr..

[57] Minor W., Cymborowski M., Otwinowski Z., Chruszcz M. (2006). HKL-3000: the integration of data reduction and structure solution--from diffraction images to an initial model in minutes. Acta Crystallogr. Section D, Biol. Crystallogr..

[58] Jumper J., Evans R., Pritzel A., Green T., Figurnov M., Ronneberger O. (2021). Highly accurate protein structure prediction with AlphaFold. Nature.

[59] Murshudov G.N., Skubak P., Lebedev A.A., Pannu N.S., Steiner R.A., Nicholls R.A. (2011). REFMAC5 for the refinement of macromolecular crystal structures. Acta Crystallogr. Section D, Biol. Crystallogr..

[60] Emsley P., Cowtan K. (2004). Coot: model-building tools for molecular graphics. Acta Crystallogr. Section D, Biol. Crystallogr..

[61] Adams P.D., Afonine P.V., Bunkoczi G., Chen V.B., Davis I.W., Echols N. (2010). PHENIX: a comprehensive Python-based system for macromolecular structure solution. Acta Crystallogr. Section D, Biol. Crystallogr..

[62] Winn M.D., Isupov M.N., Murshudov G.N. (2001). Use of TLS parameters to model anisotropic displacements in macromolecular refinement. Acta Crystallogr. Section D, Biol. Crystallogr..

[63] Davis I.W., Murray L.W., Richardson J.S., Richardson D.C. (2004). MOLPROBITY: structure validation and all-atom contact analysis for nucleic acids and their complexes. Nucleic Acids Res..

[64] Laskowski R.A. (2001). PDBsum: summaries and analyses of PDB structures. Nucleic Acids Res..

[65] Pettersen E.F., Goddard T.D., Huang C.C., Meng E.C., Couch G.S., Croll T.I. (2021). UCSF ChimeraX: structure visualization for researchers, educators, and developers. Protein Sci..

[66] Hopkins J.B., Gillilan R.E., Skou S. (2017). BioXTAS RAW: improvements to a free open-source program for small-angle X-ray scattering data reduction and analysis. J. Appl. Crystallogr..

[67] Gode-Potratz C.J., Kustusch R.J., Breheny P.J., Weiss D.S., McCarter L.L. (2011). Surface sensing in Vibrio parahaemolyticus triggers a programme of gene expression that promotes colonization and virulence. Mol. Microbiol..

[68] Bensadoun A., Weinstein D. (1976). Assay of proteins in the presence of interfering materials. Anal. Biochem..

[69] Li P., Kinch L.N., Ray A., Dalia A.B., Cong Q., Nunan L.M. (2017). Acute hepatopancreatic necrosis disease-causing Vibrio parahaemolyticus strains maintain an antibacterial type VI secretion system with versatile effector repertoires. Appl. Environ. Microbiol..

[70] Sievers F., Higgins D.G. (2018). Clustal Omega for making accurate alignments of many protein sequences. Protein Sci..

[71] Robert X., Gouet P. (2014). Deciphering key features in protein structures with the new ENDscript server. Nucleic Acids Res..

[72] Jana B., Fridman C.M., Bosis E., Salomon D. (2019). A modular effector with a DNase domain and a marker for T6SS substrates. Nat. Commun..

[73] Mougous J.D., Cuff M.E., Raunser S., Shen A., Zhou M., Gifford C.A. (2006). A virulence locus of Pseudomonas aeruginosa encodes a protein secretion apparatus. Science.

[74] Chung H.S., Raetz C.R. (2010). Interchangeable domains in the Kdo transferases of Escherichia coli and Haemophilus influenzae. Biochemistry.

[75] Fridman C.M., Keppel K., Gerlic M., Bosis E., Salomon D. (2020). A comparative genomics methodology reveals a widespread family of membrane-disrupting T6SS effectors. Nat. Commun..

[76] Dunn A.K., Millikan D.S., Adin D.M., Bose J.L., Stabb E.V. (2006). New rfp- and pES213-derived tools for analyzing symbiotic Vibrio fischeri reveal patterns of infection and lux expression in situ. Appl. Environ. Microbiol..

