# Supplementary figures and images for: A conserved chaperone protein is required for the formation of a noncanonical type VI secretion system spike tip complex
Source: J Biol Chem. 2025 Jan 27;301(3):108242. doi: 10.1016/j.jbc.2025.108242 (PMC11883445; doi:10.1016/j.jbc.2025.108242)

Figure S1

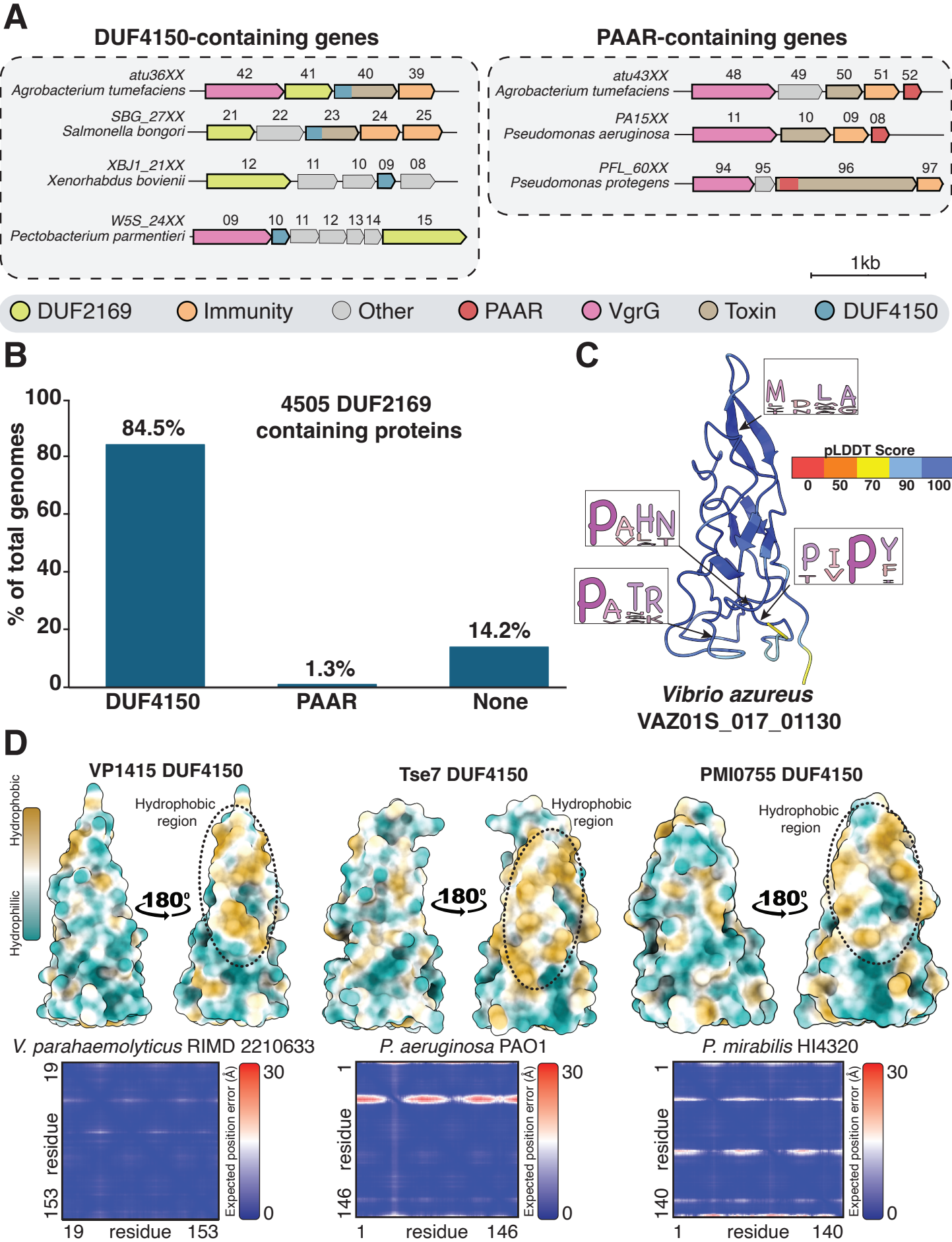

Supplement: Fig. S1 [file mmc1.pdf]

Figure S2

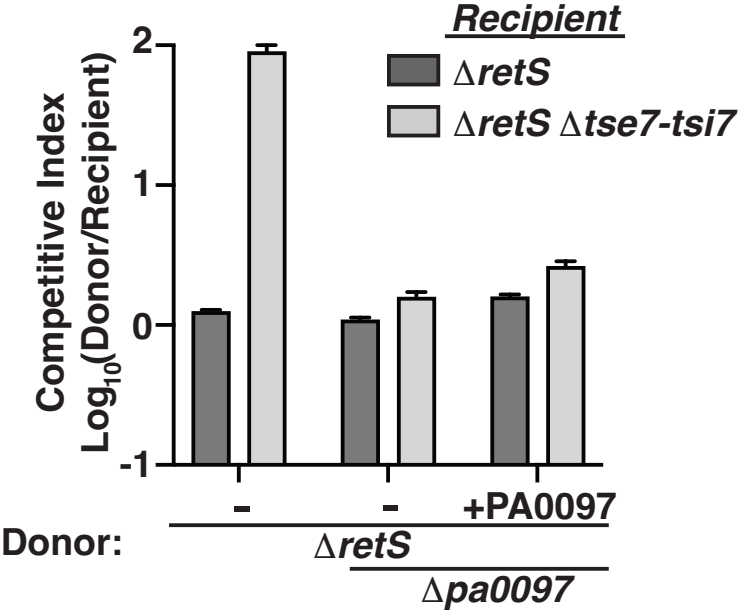

Supplement: Fig. S2 [file mmc2.pdf]

Figure S3

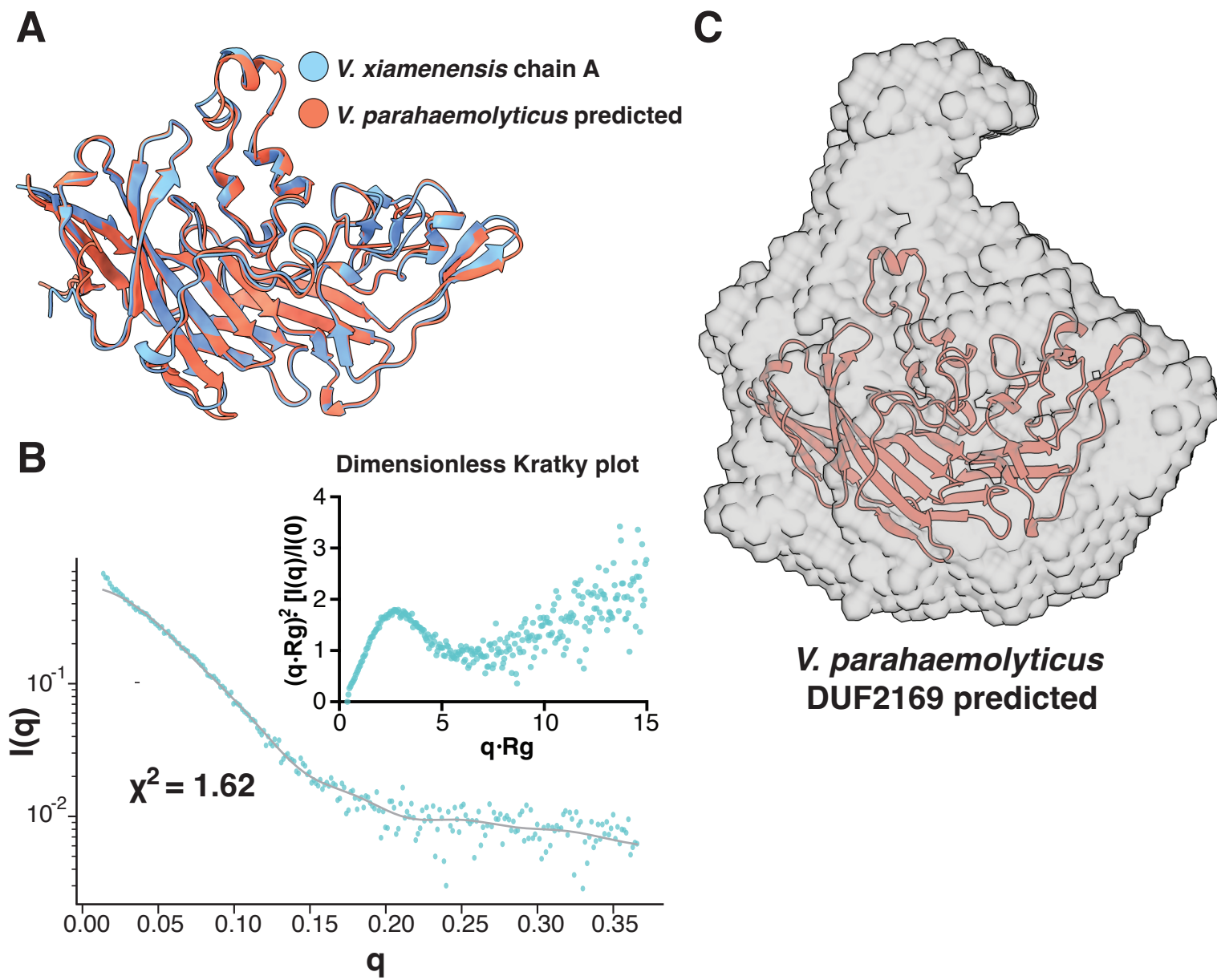

Supplement: Fig. S3 [file mmc3.pdf]

Figure S4

DUF2169-PIPY Predicted Complexes

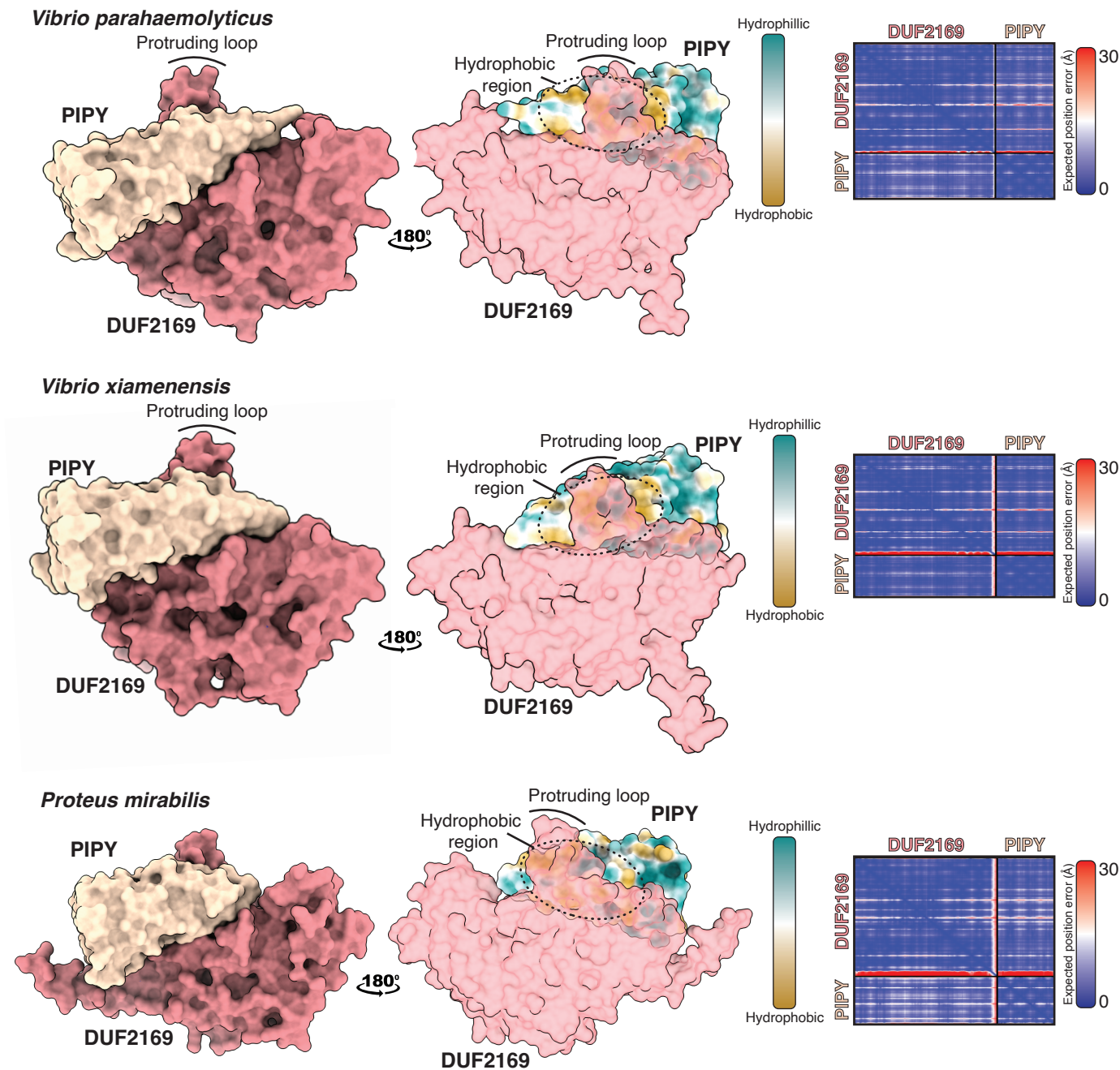

Supplement: Fig. S4 [file mmc4.pdf]
