# Supplementary material for: A conserved chaperone protein is required for the formation of a noncanonical type VI secretion system spike tip complex
Source: J Biol Chem. 2025 Jan 27;301(3):108242. doi: 10.1016/j.jbc.2025.108242 (PMC11883445; doi:10.1016/j.jbc.2025.108242)
Supplement: Supplementary Information and Data [file mmc5.docx]

**Figure S1. A) Genomic context and structural comparison of DUF4150 ‘PAAR-like’ and PAAR domains.** A) Genomic organization of DUF4150 PAAR-like (PIPY) and PAAR-containing genes indicates that these domains are encoded at the N-terminus of T6SS toxins or as single domain proteins. B) Frequency at which genes encoding PAAR or DUF4150 (PIPY) domains are located near a gene encoding a DUF2169 domain in the same genome across 4505 surveyed genomes. C) pLDDT score of AF3 *V. azureus* VAZ01S_017_01130 (residues 1-137) indicates a high confidence prediction of the PAAR-like (PIPY) domain. D) AF3 structure predictions of PAAR-like (PIPY) domains highlight the presence of a conserved hydrophobic face across different species, with confidence scores indicated below.

**Figure S2*.* DUF2169 is necessary for Tse7-dependent interbacterial in *P. aeruginosa*.** A)

Results of intraspecies growth competition assays between the indicated *P. aeruginosa* PAO1 donor and recipient strains. Donor strains were competed with recipient strains lacking *tse7-tsi7*. All deletion strains were derived using same parental Δ*retS* strain to activate type VI secretion. Data are mean ± s.d. for *n* = 3 biological replicates. Attempted gene complementation was performed using pPSV39 encoding *pa0097* with a C-terminal VSV-G tag.

**Figure S3.** **SAXS molecular envelope of *V. parahaemolyticus* DUF2169**. A) A structural alignment of the AF3 prediction of DUF2169 from *V. parahaemolyticus* (VP1398, orange) and chain A of the X-ray data derived DUF2169 structure from *V. xiamenensis* (blue) shows that they are nearly identical with a Cα RMSD of 0.69Å. B) Experimental SAXS data for *V. parahaemolyticus* DUF2169 (VP1398) and its associated P(r) distribution plot and dimensionless Kratky plot, with the latter indicating a partially unfolded protein in solution. (C) The SAXS-derived ab initio envelope fitted with the AF3-predicted structural model of *V. parahaemolyticus* DUF2169 (VP1398).

**Figure S4.** **AlphaFold3 predictions of the complexes formed by the indicated cognate DUF2169-PIPY pairs.** *V. parahaemolyticus* DUF2169 (VP1398) with PIPY (VP1415; residues 19-153), *V. xiamenensis* DUF2169 (SAMN04488136_12145) with PIPY (SAMN04488136_12162; residues 1-137) and *P. mirabilis* DUF2169 (PMI0753) with PIPY (PMI0755; residues 1-140).

**Table S1. List of bacterial strains used in this study.**

| **Strain name** | **Genotype** | **Comments** | **Source** |
| --- | --- | --- | --- |
| *Vibrio parahaemolyticus* RIMD 2210633 | Wild-type | Used for microscopy, secretion, and competition assays; also used as a template for PCR amplifications | Obtained from Kim Orth |
| *Vibrio parahaemolyticus* RIMD 2210633 Δ*hcp1* | Δ*vp_rs06770* | RIMD derivative with an in-frame deletion of *hcp1* (WP_005480650.1); used for microscopy, secretion, and competition assays | (72) |
| *Vibrio parahaemolyticus* RIMD 2210633 Δ*vp1398* | Δ*vp_rs06790* | RIMD derivative with an in-frame deletion of *VP_RS06790* (VP1398, WP_005480591.1); used for microscopy, secretion, and competition assays | This study |
| *Vibrio natriegens* ATCC 14048 | Wild-type | Used as prey in bacterial competition assays | ATCC collection |
| *Escherichia coli* DH5α (λ pir) | K-12 derivative laboratory strain containing λ pir | Used for plasmid maintenance, cloning, and tri-parental mating | Obtained from Eric V. Stabb |
| *Escherichia coli* XL-1 Blue |  | Used for plasmid maintenance and cloning |  |
| *Escherichia coli* CodonPlus (DE3) |  | Used for heterologous protein expression. |  |
| *Escherichia coli* SM10 | K-12 derivative laboratory strain containing λ pir | Used for conjugating plasmids into *Pseudomonas aeruginosa* PAO1 |  |
| *Pseudomonas aeruginosa* PAO1 Δ*retS* | Δ*retS* | Used for competition assays; used as template for further deletions | (73) |
| *Pseudomonas aeruginosa* PAO1 Δ*retS* Δ*tse7-tsi7* | Δ*retS* Δ*tse7-tsi7* | Used for competition assays. | This study |
| *Pseudomonas aeruginosa* PAO1 Δ*retS* Δ*pa0097* | Δ*retS* Δ*pa0097* | Used for competition assays. | This study |

**Table S2. List of primers used in this study.**

| **Primer name** | **Sequence (5’ to 3’)^a, b, c^** | **Description** |
| --- | --- | --- |
| pBAD33.1_GIB_F | **GATTACAAGGATGACGACGATAAG**TGAAAGCTTGGCTGTTTTGGCGG | Used to amplify the pBAD33.1^F^ plasmid backbone for Gibson assembly |
| pBAD33.1_GIB_R | CATATGTATATCTCCTTCTTAAAGTTAAACAAAATTATTTCTAGAG |  |
| VP1398_pBAD33.1_F | ctttaagaaggagatatacatATGCAGTTATGGGATATTGAAGCG | Used to amplify CDS of *VP_RS06790* (WP_005480591.1) to construct pVP1398 |
| VP1398_pBAD33.1_NoStop_R | cgtcgtcatccttgtaatcTGCATGTTCGCTTAC |  |
| VP1398_F209A_F | CATTGGCTGGTACGGCTGACGAAGAATGGATTG | Used to amplify CDS of *VP_RS06790* (WP_005480591.1) to construct pVP1388^F209A^ |
| VP1398_F209A_R | CAATCCATTCTTCGTCAGCCGTACCAGCCAATG |  |
| VP1398_W213A_F | GTTTGACGAAGAAGCGATTGAGAACCGCAAG | Used to amplify CDS of *VP_RS06790* (WP_005480591.1) to construct pVP1388^W213A^ |
| VP1398_W213A_R | CTTGCGGTTCTCAATCGCTTCTTCGTCAAAC |  |
| pDM4_Gib_F | tactcgagggtcgacggtatcgataagcttgatatacactcc | Used to amplify the pDM4 plasmid backbone for Gibson assembly |
| pDM4_Gib_R | tactcgagggtcgacggtatcgataagcttgatatacactcc |  |
| VP1398_UP_pDM4_Gib_F | gagctcaggttacccTTGGTATTTTTCATCTTCCACC | Used to amplify 600 bp upstream of *VP_RS06790* to construct pDM4 Δ*vp1398* |
| VP1398_UP_pDM4_Gib_R | CCCAGCACGTAACGACCTGTTGAGTTATGCATTCAATATCCCATAACTGCATTGCTATTCCTTAAATTG |  |
| VP1398_DN_pDM4_Gib_F | CAATTTAAGGAATAGCAATGCAGTTATGGGATATTGAATGCATAACTCAACAGGTCGTTACGTGCTGGG | Used to amplify 600 bp downstream of *VP_RS06790* to construct pDM4 Δ*vp1398* |
| VP1398_DN_pDM4_Gib_R | gtcgaccctcgagtaAAGTTGCGCTAAAGG |  |
| 5'_PA0097_R | TTCAGCATGCTTGCGGCTCGAGTTTCAGTTGATCTGCACCGAACCG | Used to amplify 500 bp downstream of *pa0097* to construct pGEX2: *Δpa0097* |
| 3'_PA0097_F | AACTCGAGCCGCAAGCATGCTGAAATGAGCCAGGCCCTGAGCATC |  |
| 5'_PA0097_F | TACGAAAGCTTGGCAGCATCGTCATCAGCGG |  |
| 3'_PA0097_R | CTAGAGAATTCGGAATGAAGCCGTTGGAGTTCTG |  |
| PA0100_DN_F | TATACAGATATTGAAATGAATAGATTAGGAAAACGCATAGATTGAGCATCGCAGCG | Used to amplify 500 bp downstream of *pa0099-0100* to construct pGEX2: *Δtse7-tsi7* |
| PA0100_DN_R | GATGCGGATCCGAATATTCGCCATCAGGTCGCGC |  |
| PA0099_UP_F | ATTCGAAGCTTGGCGTCGACTCCTTCGTCAGC |  |
| PA0099_UP_R | TTTTCCTAATCTATTCATTTCAATATCTGTATACATCAGGCAGCCCTCACCC |  |
| PA0097_RBS_SacI_F | GATGCGAGCTCACGGGAGGAAAGGTGGCGCCTATCTTTCCAGC | Used to amplify *pa0097* to construct pPSV39:*pa0097* |
| PA0097_XbaI_R | GCTAGTCTAGATTCATTTTCCTAATCTATTCATTTCAATATCTGTATACATGCCTCGTCCTCCTCGG |  |
| VP1398_NdeI_F | GCTACCATATGCAGTTATGGGATATTGAAGCGTACC | Used to amplify *vp1398* to construct pET29b:*vp1398* |
| VP1398_XhoI_R | GATCACTCGAGTGCATGTTCGCTTACCTCTTCCAC |  |
| VP1398_XhoI_VSVG_R | **GATCACTCGAGTCATTTTCCTAATCTATTCATTTCAATATCTGTATA**TGCATGTTCGCTTACCTCTTCCAC | Used to amplify *vp1398* to construct pET29b:*vp1398-VSV-G* |
| VP1415_DUF4150_XhoI_R | GACGTCTCGAGTCAACACATAGTGTTGGCTTTGTTCATGG | Used to amplify *vp1415(19-154)* to construct pETDuet-1:*empty*:FLAG*-vp1415(1-154)* |
| FLAG_VP1415_NdeI_F | **GACGTCATATGGACTATAAGGATGATGATGATAAG**GGCGTAACAGTTGGTGCAAATGG |  |

^a^ Uppercase letters denote to gene sequences; lowercase letters denote plasmid sequences

^b^ Underlined letters denote sequence changes leading to point mutations

^c^ Bold letters denote tag sequence

**Table S3. List of plasmids used in this study.**

| **Plasmid name** | **Description** | **Purpose** | **Source** |
| --- | --- | --- | --- |
| pEmpty | pBAD33.1 mobilizable plasmid for arabinose-inducible protein expression, with ori15A and Cm^R^ | Used as a control for the arabinose-inducible expression of proteins in *V. parahaemolyticus* RIMD 2210633, and as a template to generate pBAD33.1^F^ | (74) |
| pBAD33.1^F^ | pEmpty with a FLAG tag inserted between the first methionine codon following the ribosome binding site (RBS) and the HindIII restriction site within the MCS | Used as the vector backbone for the construction of arabinose inducible expression plasmids for *Vibrio parahaemolyticus* | (75) |
| pVP1398 | pBAD33.1^F^ plasmid containing CDS of VP1398 (WP_005480591.1), in frame with the C-terminal FLAG tag | Used for the arabinose-inducible expression of VP1398 in *V. parahaemolyticus* RIMD 2210633 | This study |
| pVP1398^F209A^ | pVP1398 containing a substitution of phenylalanine 209 with alanine | Used for the arabinose-inducible expression of VP1398^F209A^ in *V. parahaemolyticus* RIMD 2210633 | This study |
| pVP1398^W213A^ | pVP1398 containing a substitution of with a substitution of tryptophan 213 with alanine | Used for the arabinose-inducible expression of VP1398^W213A^ in *V. parahaemolyticus* RIMD 2210633 | This study |
| pVSV209 | dsRed and Kan^R^ cassette-containing plasmid | Used for selective growth of *V. natriegens* prey in competition assays | (76) |
| pDM4 | Suicide plasmid harboring *sacB* gene encoding levansucrase (*SacB*), Cm^R^ and R6K ori | Used as a template to amplify the plasmid backbone for subsequent cloning | (52) |
| pDM4:Δ*vp1398* | pDM4 with the sequences 600 bp upstream and downstream of *VP_RS06790* cloned into its MCS | Used for deletion of *VP_RS06790* in *V. parahaemolyticus* RIMD 2210633 | This study |
| pTssB1‐sfGFP | pBAD/Myc-His plasmid containing the CDS of TssB1 (WP_005480589.1) fused with sfGFP, in frame with the C-terminal Myc tag | Used in microscopy to visualize T6SS1 sheath assembly | (39) |
| pEXG2::Δ*pa0097* | Suicide plasmid harboring 500 bp upstream and downstream of *pa0097 to generate in-frame gene deletion* | Used to generate *pa0097* gene deletion | This study |
| pEXG2::Δ*tse7-tsi7* | Suicide plasmid harboring 500 bp upstream and downstream of *pa0099-0100 to generate in-frame gene deletion* | Used to generate *pa0099-0100* gene deletion | This study |
| pPSV39 | IPTG-inducible protein expression, with oriT and gentamicin resistant cassette | Used for empty vector control | (9) |
| pPSV39-CV::*pa0097-VSV-G* | Encodes IPTG-inducible *pa0097* gene with a C-terminal  VSV-G tag | Used for complementation of PA0097 in *P. aeruginosa* PAO1 | This study |
| pET29b::*SAMN04488136_12145-*his6 | IPTG-inducible vector with kanamycin resistant cassette encoding *V. xiamenensis* DUF2169 with C-terminal His6 tag | Used for heterologous expression of *V. xiamenensis* DUF2169 | Synthesized by Genscript. |
| pET29b::*vp1398-*his6 | IPTG-inducible vector with kanamycin resistant cassette encoding *V. parahaemolyticus* DUF2169 with C-terminal His6 tag | Used for heterologous expression of *V. parahaemolyticus* DUF2169 | This study |
| pET29b::*vp1398-*VSV-G | IPTG-inducible vector with kanamycin resistant cassette encoding *V. parahaemolyticus* DUF2169 with C-terminal VSV-G tag | Used for pulldown assay with *V. parahaemolyticus* PIPY | This study |
| pETDuet-1::*empty* ::FLAG*-vp1415(1-154)* | IPTG-inducible vector with ampicillin resistant cassette encoding *parahaemolyticus* PIPY with N-terminal FLAG tag | Used for pulldown assay with *V. parahaemolyticus* DUF2169 | This study |

**Supplementary Dataset S1.** List of DUF2169-containing proteins in bacterial genomes.

**Supplementary Dataset S2.** List of DUF4150-containing proteins in bacterial genomes.

**Supplementary Dataset S3.** Bacterial genomes containing DUF2169 and/or DUF4150.
